# Wine consumption, Mediterranean diet, and cardiovascular risk in two Spanish cohorts

**DOI:** 10.1093/eurheartj/ehaf1081

**Published:** 2026-02-11

**Authors:** Miguel A Martínez-González, Maira Bes-Rastrollo, Carmen Sayon-Orea, Miguel Ruiz-Canela, Juan Timiraos, Estefanía Toledo, José V Sorlí, Jordi Salas-Salvado, Enrique Gómez-Gracia, Miquel Fiol, Helmut Shröeder, José Lapetra, Luis Serra-Majem, Xavier Pintó, María Barbería-Latasa, Emilio Ros, Nancy Babio, Carolina Ortega, Rafael de la Torre, Rosa M Lamuela-Raventos, Ramón Estruch

**Affiliations:** Instituto de Salud Carlos III, Centro de Investigación Biomédica en Red Fisiopatología de la Obesidad y Nutrición (CIBERObn), Madrid, Spain; Department of Preventive Medicine and Public Health, University of Navarra, Pamplona, Spain; Navarra Institute for Health Research (IdiSNA), Pamplona, Spain; Department of Nutrition, Harvard T.H. Chan School of Public Health, Boston, USA; Instituto de Salud Carlos III, Centro de Investigación Biomédica en Red Fisiopatología de la Obesidad y Nutrición (CIBERObn), Madrid, Spain; Department of Preventive Medicine and Public Health, University of Navarra, Pamplona, Spain; Navarra Institute for Health Research (IdiSNA), Pamplona, Spain; Instituto de Salud Carlos III, Centro de Investigación Biomédica en Red Fisiopatología de la Obesidad y Nutrición (CIBERObn), Madrid, Spain; Department of Preventive Medicine and Public Health, University of Navarra, Pamplona, Spain; Navarra Institute for Health Research (IdiSNA), Pamplona, Spain; Instituto de Salud Carlos III, Centro de Investigación Biomédica en Red Fisiopatología de la Obesidad y Nutrición (CIBERObn), Madrid, Spain; Department of Preventive Medicine and Public Health, University of Navarra, Pamplona, Spain; Navarra Institute for Health Research (IdiSNA), Pamplona, Spain; Stroke Unit, Neurology Department, Integrated Health Organization Araba, Vitoria-Gasteiz, Spain; Instituto de Salud Carlos III, Centro de Investigación Biomédica en Red Fisiopatología de la Obesidad y Nutrición (CIBERObn), Madrid, Spain; Department of Preventive Medicine and Public Health, University of Navarra, Pamplona, Spain; Navarra Institute for Health Research (IdiSNA), Pamplona, Spain; Instituto de Salud Carlos III, Centro de Investigación Biomédica en Red Fisiopatología de la Obesidad y Nutrición (CIBERObn), Madrid, Spain; Department of Preventive Medicine, University of Valencia, Valencia, Spain; Instituto de Salud Carlos III, Centro de Investigación Biomédica en Red Fisiopatología de la Obesidad y Nutrición (CIBERObn), Madrid, Spain; Human Nutrition Unit, Faculty of Medicine and Health Sciences, IISPV (Institut d'Investigació Sanitària Pere Virgili), Rovira i Virgili University, Reus, Spain; Department of Preventive Medicine, Instituto de Investigación Biomédica de Málaga (IBIMA), University of Malaga, Málaga, Spain; Platform for Clinical Trials, Instituto de Investigación Sanitaria Illes Balears (IdISBa), Hospital Universitario Son Espases, Palma de Mallorca, Spain; Stroke Unit, Department of Neurology, Neurovascular Research Group, Institut Hospital del Mar d'Investigacions Mèdiques (IMIM), Barcelona, Spain; Instituto de Salud Carlos III, Centro de Investigación Biomédica en Red Fisiopatología de la Obesidad y Nutrición (CIBERObn), Madrid, Spain; Department of Family Medicine, Research Unit, Distrito Sanitario Atención Primaria Sevilla, Sevilla, Spain; Instituto de Salud Carlos III, Centro de Investigación Biomédica en Red Fisiopatología de la Obesidad y Nutrición (CIBERObn), Madrid, Spain; Nutrition Research Group, Research Institute of Biomedical and Health Sciences (IUIBS), University of Las Palmas de Gran Canaria, Las Palmas de Gran Canaria, Spain; Instituto de Salud Carlos III, Centro de Investigación Biomédica en Red Fisiopatología de la Obesidad y Nutrición (CIBERObn), Madrid, Spain; Lipids and Vascular Risk Unit, Internal Medicine, Hospital Universitario de Bellvitge, Hospitalet de Llobregat, Barcelona, Spain; Instituto de Salud Carlos III, Centro de Investigación Biomédica en Red Fisiopatología de la Obesidad y Nutrición (CIBERObn), Madrid, Spain; Department of Preventive Medicine and Public Health, University of Navarra, Pamplona, Spain; Navarra Institute for Health Research (IdiSNA), Pamplona, Spain; August Pi i Sunyer Biomedical Research Institute (IDIBAPS), Hospital Clinic, University of Barcelona, Barcelona, Spain; Instituto de Salud Carlos III, Centro de Investigación Biomédica en Red Fisiopatología de la Obesidad y Nutrición (CIBERObn), Madrid, Spain; Human Nutrition Unit, Faculty of Medicine and Health Sciences, IISPV (Institut d'Investigació Sanitària Pere Virgili), Rovira i Virgili University, Reus, Spain; Instituto de Salud Carlos III, Centro de Investigación Biomédica en Red Fisiopatología de la Obesidad y Nutrición (CIBERObn), Madrid, Spain; Department of Preventive Medicine, University of Valencia, Valencia, Spain; Instituto de Salud Carlos III, Centro de Investigación Biomédica en Red Fisiopatología de la Obesidad y Nutrición (CIBERObn), Madrid, Spain; Faculty of Experimental and Health Sciences, Universitat Pompeu Fabra (UPF), Barcelona, Spain; Instituto de Salud Carlos III, Centro de Investigación Biomédica en Red Fisiopatología de la Obesidad y Nutrición (CIBERObn), Madrid, Spain; Grup de Recerca Antioxidants Naturals: Polifenols, Departament de Nutrició, Ciencies de l’Alimentació i Gastronomia, Facultat de Farmacia, Universitat de Barcelona, Barcelona, Spain; Institut de Recerca en Nutrició i Seguretat Alimentària, Universitat de Barcelona (INSA-UB), Barcelona, Spain; Instituto de Salud Carlos III, Centro de Investigación Biomédica en Red Fisiopatología de la Obesidad y Nutrición (CIBERObn), Madrid, Spain; August Pi i Sunyer Biomedical Research Institute (IDIBAPS), Hospital Clinic, University of Barcelona, Barcelona, Spain; Institut de Recerca en Nutrició i Seguretat Alimentària, Universitat de Barcelona (INSA-UB), Barcelona, Spain; Department of Medicine, Hospital Clinic, Carrer Villarroel, 170, Barcelona 08036, Spain; Institut d'Investigacions Biomèdiques Agust Pi i Sunyer (IDIBAPS), Barcelona, Spain; School of Medicine, University of Barcelona, Barcelona, Spain

**Keywords:** Wine, Cardiovascular disease, Mediterranean Diet, Mortality, PREDIMED trial, Stroke

## Abstract

**Background and Aims:**

The benefits of the Mediterranean diet (MedDiet) are well established. However, one component, wine, remains controversial. This study assessed the association between MedDiet (with or without wine consumption) and major cardiovascular disease (CVD) or all-cause mortality.

**Methods:**

The PREDIMED trial included 7447 high-risk participants. Adherence to MedDiet was measured using a validated 14-item questionnaire, including one item on wine (cut-off: seven glasses/week). The CVD events were recorded over a 4.8-year follow-up, while all-cause mortality was tracked for 17 years. A younger Spanish cohort (the SUN project), including 23,133 participants followed up for 22 years, was also evaluated.

**Results:**

In PREDIMED, compared with poor compliers with MedDiet (excluding wine), good compliers (excluding wine), had a multivariable-adjusted hazard ratio (HR) of 0.84 [95% confidence interval (CI) 0.61–1.15] for CVD. For good compliers with MedDiet (including wine), the HR for CVD was 0.55 (95% CI 0.36–0.83). For all-cause mortality, MedDiet compliers (excluding wine) had HR of 0.77 (95% CI 0.68–0.87), which was 0.67 (95% CI 0.57–0.78) for MedDiet compliers (including wine). In exploratory dose–response analyses, reduced risk for death was not present in PREDIMED participants who drank three or more glasses of wine/day. Additionally, analyses least vulnerable to threats of abstainer bias were not significant and neither were multiplicative interaction terms for the wine item in the questionnaire. In the SUN cohort, no significant associations were observed between MedDiet compliance, wine, and CVD. However, for all-cause mortality, the HR was 0.94 (95% CI 0.71–1.26) for MedDiet compliers (excluding wine) and 0.54 (95% CI 0.28–1.04) for MedDiet compliers (including wine). When pooling both cohorts, wine consumption within the MedDiet was associated with lower all-cause mortality (*P* = .01).

**Conclusions:**

In PREDIMED, moderate wine consumption, as part of the MedDiet, appeared to be associated with lower mortality and CVD risk. Some non-significant associations and interactions advise caution in interpretation of these findings.

## Structured Graphical Abstract

**Structured Graphical Abstract:**
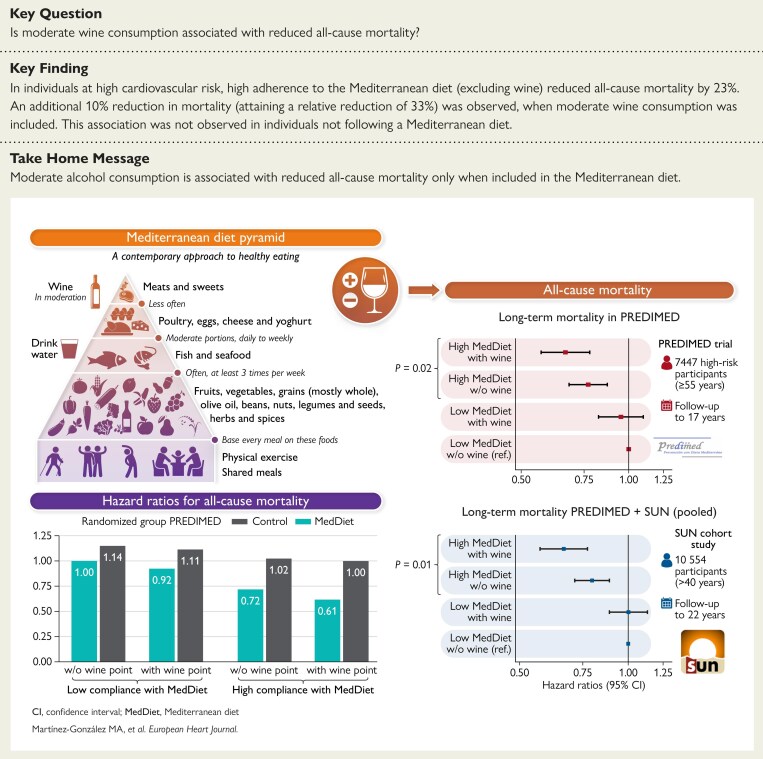

**See the editorial comment for this article ‘Does daily wine consumption add health benefits to the Mediterranean diet? Some grounds for scepticism', by T. Stockwell and J. Zhao, https://doi.org/10.1093/eurheartj/ehag253.**

## Introduction

The “PREvencion con DIeta MEDiterránea” (PREDIMED) trial reported a 30% reduction in major cardiovascular disease (CVD) incidence with a Mediterranean diet (MedDiet) compared with a low-fat control diet after 4.8-year intervention.^1^ Per-protocol analyses suggested that the benefit could be even greater with higher adherence.^1^ These findings are further supported by numerous prospective observational studies confirming the protection by the MedDiet.^2^

One of the distinguishing components of the MedDiet is moderate consumption of wine, typically one glass per day with meals,^3^ and it is formally included in the validated “Mediterranean Diet Adherence Screener” (MEDAS).^4^ High MEDAS scores, including wine consumption, have been linearly associated with reduced CVD risk, a relationship also confirmed using plasma metabolomic signatures.^5^

However, the role of wine within cardioprotective dietary patterns remains controversial.^6–9^ Beyond CVD, the assessment of all-cause mortality as an outcome is crucial, as the adverse effects of ethanol on cancer risk could potentially counteract cardiovascular benefits of moderate wine consumption. In this context, some cohort studies found that removing alcohol from the MedDiet score attenuated its inverse association with both all-cause mortality^10,11^ and CVD incidence.^12^ Nevertheless, these observational findings,^13^ including those relying on genetic proxies for alcohol use,^8,14^ may be limited by residual confounding or other methodological shortcomings. Beyond the quantity of alcohol, both the type of beverage and the drinking pattern (e.g. moderate daily consumption with meals) may act as key effect modifiers.^15–20^

In the absence of large randomized trials specifically evaluating alcohol consumption,^21–23^ it would be valuable to assess the impact of moderate wine consumption within the context of a MedDiet randomized intervention,^24^ particularly when repeated dietary measurements are available over time.^3^

This study aims to assess the association between adherence to the MedDiet with or without moderate wine consumption and both major CVD events and all-cause mortality in the PREDIMED trial. These associations were also assessed in the “Seguimiento University of Navarra” (SUN) project, a younger Spanish cohort with high long-term follow-up retention and comparable dietary assessment methodology.

## Material and methods

### Data sources including study covariates

Two well-known prospective studies were used as data sources, namely, the PREDIMED trial^1,25^ and the SUN cohort study.^12,26^ Briefly, PREDIMED was conducted at 11 Spanish centres where participants were randomized in a 1:1:1 ratio to one of three dietary interventions: MedDiet supplemented with extra-virgin olive oil, MedDiet supplemented with mixed nuts, or low-fat control diet. The trial evaluated the intention-to-treat effects of these dietary interventions on CVD over a median follow-up of 4.8 years. A total of 7447 participants were randomized, comprising 4282 women (aged 60–80 years) and 3165 men (aged 55–80 years) without CVD at baseline, but at high cardiovascular risk. All participants received quarterly face-to-face nutritional counselling delivered by trained dietitians. The trial protocol was approved by the Institutional Review Boards (IRB) at all study sites in Spain and was registered at Current Controlled Trials (ISRCTN35739639). Covariates collected in PREDIMED have been described previously.^27,28^ At the initial screening, participants self-reported data on lifestyle factors, family history of diseases, medical history, and medication use. Anthropometric measurements were recorded by trained personnel and fasting blood samples were collected to assess lipids, glucose, and other biochemical markers at baseline. All covariate measurements, including anthropometry, biochemistry, and dietary assessments (see below), were repeated annually throughout the trial period.

The SUN cohort study was used as a data source to complement the findings of PREDIMED. SUN is a prospective, dynamic cohort of Spanish university graduates, initiated in December 1999 and permanently open to recruitment, as previously described.^26^ For the present analysis, the database completed in May 2022, which included 23 133 Spanish university graduates, was used. The SUN protocol was developed under the supervision of the Harvard T.H. Chan School of Public Health, approved by the University of Navarra IRB and registered at ClinicalTrials.gov (NCT02669602). Baseline and follow-up data were collected in the SUN cohort using self-administered questionnaires, which gathered information on sociodemographic characteristics, health status, family history of disease, and lifestyle information, including dietary intake. Follow-up questionnaires were sent biennially to update this information, including medication use and clinical outcomes. The validity of self-reported information in this highly educated cohort was confirmed through specific validation studies.^26^ For the present analyses, participants younger than 40 years during the first 5-year follow-up, those who had less than 2 years and 9 months of follow-up, and those lost to follow-up (overall retention rate: 96.5%) were excluded. After exclusions, the final sample included 10 554 participants.

### Study exposures

Adherence to the MedDiet was assessed annually in PREDIMED using the validated 14-item MEDAS questionnaire, which scored from 0 (lowest adherence) to 14 (highest adherence). Interviews were conducted by trained dietitians.^4,28–30^ One point was awarded for each of the following criteria: use of olive oil as main culinary fat; daily consumption of olive oil (≥4 servings/day); preference of white meat over red/processed meat; high consumption of vegetables (≥2 servings/day), fruit (≥3 servings/day), and legumes (≥3 servings/week); fish/seafood (≥3 servings/week); tree nuts (≥3 servings/week); and ‘sofrito’ (a sauce made with tomato, garlic, onion or leeks, sautéed in olive oil) (≥2 times/week). Additionally, one point was awarded for low consumption of red meat (<1 serving/day); butter, margarine, or cream (<1 serving/day); sugar-sweetened beverages (<1 cup/day); and commercial pastries (<2 servings/week). Lastly, moderate weekly consumption of specifically wine (≥7 glasses/week) was also scored with one point and served as one of the two components of the main exposure in the present analyses. Repeated MEDAS data were collected annually by the dietitians, from baseline through up to 7 years of follow-up.

Both PREDIMED and SUN applied a validated semi-quantitative food-frequency questionnaire (FFQ) to obtain information on dietary habits and alcohol intake.^31^ This questionnaire was used in the SUN cohort to derive the MEDAS score^12^ and to quantify total alcohol intake (g/day) and wine consumption (mL/day), as previously described.^15,31^ It was collected at baseline and after 10-year follow-up in the SUN cohort. The validated FFQ and the MEDAS were repeatedly obtained at baseline and on every follow-up year during the PREDIMED trial (up to 7 years). In PREDIMED, the last date of alcohol consumption prior to study inception was collected by dietitians at baseline and this information was used in combination with the alcohol intake data from the FFQ to build the category of former drinkers.

### Study outcomes

The CVD events (myocardial infarction, stroke, or cardiovascular deaths) were the components of the primary endpoint in the PREDIMED trial.^1,25,28^ They were adjudicated by the Clinical End-Point Adjudication Committee through 1 December 2010. This Committee annually reviewed participants’ medical records to confirm events, blinded to group allocation, dietary adherence, and wine consumption. Mortality was assessed in PREDIMED through a comprehensive process that included annual review of medical records, repeated direct follow-ups of participants and next of kin, and yearly consultation of the Spanish National Death Index. Data were validated by the same End-Point Adjudication Committee. Complete all-cause mortality follow-up was available for PREDIMED through 31 December 2020, via National Death Index from the Spanish Institute of Statistics.

The main outcomes assessed in the SUN cohort were also CVD events (myocardial infarction, stroke, or cardiovascular deaths) and all-cause mortality. Although a prior study evaluated the association of MedDiet (including or not alcohol) on CVD within the SUN cohort,^12^ the present analysis incorporates updated and long-term mortality data. The CVD events were confirmed, as previously reported,^12,26^ through review of medical records by trained cardiologists, who were masked with respect to compliance with the MedDiet and to wine consumption. Deaths were confirmed in the SUN cohort using death certificates, medical records, and linkage with the Spanish National Institute of Statistics. The National Death Index was consulted annually, and cause-of-death verification was recorded through January 2022.

### Statistical analyses (main analyses, subgroup analyses, and sensitivity analyses)

The PREDIMED study examined the association between compliance with the MedDiet (with or without wine) and the risk of CVD (during the trial period; median follow-up of 4.8 years) or all-cause mortality (both during the trial and over an extended follow-up of 17 years). Analyses were conducted using time-varying multivariable Cox regression models, incorporating yearly cumulative averages of time-dependent variables up to each participant’s most recent visit. The robust estimate of variance proposed by Huber and White was used in all models. The main exposure was the joint classification according to the attainment (no/yes) of the wine point from the MEDAS score, combined with cumulative averages of the other 13 MEDAS items (≤ 9 vs >9–13 points). Cumulative averages were calculated using up to eight time points (baseline + up to 7-year annual follow-ups). Using as reference group the participants with ≤9 points in the 13-item MEDAS and 0 wine point, three hazard ratios (HRs) were estimated for the following categories: (i) ≤9 MEDAS + 1 wine point; (ii) >9 MEDAS and 0 wine point; and (iii) >9 MEDAS + 1 wine point. Multivariable Model 1 was adjusted for age and sex, without any stratifying variables. Multivariable Model 2 was adjusted for age, smoking status (never/current/former), diabetes, hypertension, dyslipidaemia, physical activity (continuous, METS-h/week), waist-to-height ratio (continuous), body mass index (continuous kg/m^2^, including a quadratic term), total energy intake (continuous, kcal/day), fruit consumption (continuous, g/day), vegetable consumption (continuous, g/day), and dietary fibre intake (continuous, g/day). Model 2 was stratified by sex, site, educational level (five categories), and randomization arm. Subgroup analyses were conducted separately for men and women and for non-smokers. As an ancillary analysis, separate fully adjusted models were also run comparing the category with >9 points in the 13-item MEDAS and attainment of the wine point vs >9 points in 13-item MEDAS and no wine point (new reference category). This last comparison allowed to appraise the add-on association of wine beyond the inverse association observed for high adherence to the MedDiet.

For the absolute mortality risks in PREDIMED during the extended follow-up periods, cumulative mortality risks over 17 years were plotted using inverse-probability weighting (IPW) with categories defined by the baseline data. A multinomial logistic regression adjusted for the above covariates was used to obtain the probabilities of baseline exposures to be applied for the weights. Similarly, absolute CVD risks were plotted for each of the four categories of the joint exposure to dichotomized baseline 13-MEDAS (≤9 or >9) and the baseline wine point (yes/no) for the trial period (2003–10) using IPW. Finally, the HRs for mortality were also graphically represented. First, a four-category classification combining compliance with the 13-item MEDAS and the wine point was used: poor complier (≤9) and no wine point (code = 0); poor complier with MedDiet (≤9) but attainment of the wine point (code = 1); good complier with MedDiet (>9 points) and no wine point (code = 2); and good complier with MedDiet (>9 points) and the wine point (code = 3). Linear trend tests across these four categories were conducted using values from 0 to 3 as a continuous variable.

Then, this four-group classification was combined with the randomized intervention (MedDiet/control), thus building eight exposure categories. Using low-adherence, no wine, and randomization to the two MedDiet groups as the reference category, HRs for the other seven exposure combinations were estimated after adjusting for the confounders previously mentioned as Multivariable Model 2.

In the SUN cohort, similar models were applied for total mortality, including the use of repeated dietary measurements. A difference was that in the SUN cohort, only two dietary measurement time points (baseline and 10-year follow-up) were available. Despite fewer time points, the SUN cohort had a considerably longer follow-up (22 years) for this younger, lower-risk population. The younger age, lower risk—and consequently lower number of events—may lead to problems of sparse data in some exposure categories in the SUN cohort. In addition, average adherence to the MedDiet was lower in SUN than in PREDIMED. Consequently, to avoid sparse data, a slightly lower cut-off point (≥9 points instead of >9) was set for considering high cumulative average compliance with the 13-item MEDAS score. Missing data were minimal in the PREDIMED and SUN cohorts for the main variables (<5%), and their values were carried forward using data from adjacent time points. Finally, the main results from PREDIMED and SUN were pooled via fixed-effect meta-analysis. All tests were two-sided, with *P* < .05 considered statistically significant and 95% confidence intervals (CIs) were calculated for all estimates.

Subgroup analyses in PREDIMED included stratification by sex and assessments restricted to non-smokers and to only drinkers (i.e. removing abstainers, removing former drinkers, or removing both) or to those who did not substantially reduce their alcohol intake during follow-up.

Sensitivity analyses were conducted in PREDIMED using alcohol intake and wine consumption data derived from the yearly FFQs, as an alternative to MEDAS, also changing the category used as reference, and using alternative cut-off points. Also, the residual method of adjusting for total energy intake was applied as a sensitivity analysis to assess the relationship between categories of alcohol intake (or wine consumption) and all-cause mortality.

## Results

The baseline characteristics of participants, stratified by baseline compliance with the MedDiet and attainment of the wine point (yes/no), are presented in *Table 1* for the 7447 PREDIMED participants and in *Table 2* for the 10 554 SUN participants aged 40 years or older. Median wine consumption was 6.7 mL/day [interquartile range (IQR): 0–92.9 mL/day] in the PREDIMED cohort and 13.4 mL/day (IQR: 0–49.6 mL/day) in the SUN cohort. As expected, participants who attained the wine point had substantially higher average total ethanol intake, regardless of MedDiet compliance level. In PREDIMED, wine consumers had higher levels of education. In both cohorts, wine consumers had much higher prevalence of smoking, higher total energy intake, and only slightly greater levels of physical activity.

**Table 1:** Baseline characteristics of participants in the PREDIMED trial, classified jointly by adherence to Mediterranean diet (13-item MEDAS, excluding wine) and the attainment of the wine consumption at baseline

Wine consumption at baseline (≥1 glass/day)	Baseline MEDAS (excluding alcohol)			
	Low MedDiet adherence (0–9)		High MedDiet adherence (>9–13)	
	No wine	Adding wine	No wine	Adding wine
*N*	2685	1139	2564	1059
MEDAS score (without wine and including wine)	6.9 (1.2)	7.8 (1.2)	9.9 (1.0)	10.9 (1.0)
Baseline alcohol intake (g/day)	2.8 (7.3)	23.0 (18.9)	2.5 (5.6)	21.2 (16.0)
Average alcohol intake during follow-up (g/day)	3.3 (6.2)	17.8 (13.8)	2.9 (5.3)	17.3 (13.5)
Wine consumption (mL/day)	10.4 (28.9)	172.7 (143.3)	10.9 (27.1)	164.7 (132.4)
Beer consumption (mL/day)	33.1 (124.4)	87.1 (198.3)	27.0 (96.1)	69.1 (155.3)
Liquor consumption (mL/day)	0.4 (4.5)	1.6 (7.8)	0.3 (2.6)	1.0 (5.5)
Age	66.6 (6.2)	66.0 (5.9)	66.2 (6.1)	66.1 (6.3)
Body mass index	30.5 (3.9)	29.4 (3.4)	30.0 (4.1)	29.3 (3.3)
Waist circumference	101.2 (10.2)	101.8 (9.8)	99.0 (10.8)	100.5 (9.9)
Waist-to-height ratio	0.6 (0.1)	0.6 (0.1)	0.6 (0.1)	0.6 (0.1)
Baseline fasting plasma glucose (mg/dL)	125.0 (42.5)	122.6 (38.9)	123.2 (39.8)	121.5 (35.9)
Total blood cholesterol (mg/dL)	210 (37)	212 (37)	211 (37)	212 (37)
HDL cholesterol (mg/dL)	54.3 (13.9)	54.8 (13.6)	55.4 (14.2)	54.5 (14.2)
LDL cholesterol (mg/dL)	133 (38)	134 (36)	134 (37)	136 (37)
Triglycerides (mg/dL)	142 (75)	136 (70)	135 (70)	133 (69)
AST (aspartate-aminotransferase, UI/L)	44.4 (28.6)	43.8 (28.1)	45.6 (29.1)	44.4 (28.4)
ALT (alanine-aminotransferase, UI/L)	30.1 (15.0)	30.6 (16.0)	30.1 (15.1)	31.4 (26.6)
Leisure-time physical activity (METs-min/day)	195 (211)	271 (250)	222 (237)	299 (278)
Total energy intake	2146 (598)	2453 (604)	2234 (574)	2505 (597)
Education years	3.7 (2.1)	4.4 (2.6)	3.8 (2.0)	4.4 (2.5)
Baseline fruit intake (g/day)	346 (197)	326 (191)	409 (217)	397 (204)
Baseline vegetable intake (g/day)	308 (137)	303 (121)	370 (162)	363 (157)
Total dietary fibre intake (g/day)	23.8 (8.4)	23.8 (7.7)	27.4 (9.7)	27.5 (9.3)
Women	67.4%	28.1%	71.0%	31.2%
Current smokers	12.6%	23.5%	8.8%	20.3%
Former smokers	18.3%	37.6%	19.8%	38.7%
Dyslipidaemia at baseline	72.6%	70.6%	73.2%	71.2%
Hypertension at baseline	84.7%	80.4%	83.5%	78.4%
Diabetes at baseline	51.2%	46.4%	49.2%	44.0%
Family history of premature CHD	22.6%	18.4%	24.1%	21.9%
Randomly allocated to MedDiet + EVOO	32.8%	31.2%	35.4%	37.8%
Randomly allocated to MedDiet + nuts	30.2%	34.0%	33.6%	37.4%

MEDAS, Mediterranean Diet Adherence Screener Score; PREDIMED, “Prevencion con Dieta Mediterránea”; MedDiet, Mediterranean diet

**Table 2:** Baseline characteristics of participants older than 40 years in the SUN cohort, according to the joint classification by Mediterranean diet adherence (13-item MEDAS score, excluding wine) and attainment of wine point at baseline

Wine point at baseline (≥1 glass/day)	Baseline MEDAS (excluding alcohol)			
	Low MedDiet adherence (0–8)		High MedDiet adherence (9–13)	
	No wine	Adding wine	No wine	Adding wine
*N*	7890	1544	969	151
MEDAS score (without wine and including wine)	5.8 (1.5)	6.8 (1.5)	9.4 (0.6)	10.4 (0.7)
Baseline alcohol intake (g/day)	5.3 (8.0)	26.1 (19.2)	4.75 (7.6)	24.3 (14.6)
Wine consumption (mL/day)	17.8 (24.1)	206.6 (144.1)	16.3 (23.5)	201.9 (115.4)
Beer consumption (mL/day)	66.6 (139.8)	126.3 (199.8)	62.2 (122.9)	109.6 (150.4)
Liquors consumption (mL/day)	2.6 (9.4)	7.4 (19.8)	1.8 (12.8)	5.2 (13.3)
Age	46.8 (8.8)	50.0 (9.2)	49.4 (9.5)	51.8 (8.5)
Body mass index (kg/m^2^)	24.5 (3.6)	25.3 (3.4)	24.0 (3.3)	24.8 (3.0)
Leisure-time physical activity (METs-h/week)	20.4 (21.3)	22.8 (21.8)	26.6 (27.6)	32.2 (27.1)
Total energy intake (kcal/day)	2404 (782)	2591 (781)	2733 (849)	2877 (889)
Years of university education	5.3 (1.7)	5.4 (1.8)	5.1 (1.6)	5.2 (1.6)
Baseline fruit intake (g/day)	305 (267)	282 (229)	611 (413)	556 (351)
Baseline vegetable intake (g/day)	530 (338)	529 (354)	919 (574)	908 (690)
Total dietary fibre intake (g/day)	23.7 (10.8)	23.5 (10.1)	39.1 (17.1)	36.8 (13.7)
Women	53.0%	28.2%	65.7%	36.4%
Marital status (married)	74.3%	76.0%	71.8%	74.2%
Smoking pack-years	8.2 (11.9)	12.7 (14.1)	7.9 (11.6)	10.9 (13.3)
Current smokers	24.7%	29.1%	17.5%	15.2%
Former smokers	34.8%	46.0%	41.9%	58.3%
Depression at baseline	14.3%	14.6%	15.5%	13.2%
Cardiovascular disease at baseline	2.0%	4.0%	3.1%	6.0%
Cancer at baseline	3.8%	4.1%	4.6%	4.6%
Diabetes at baseline	2.6%	4.0%	2.7%	3.3%
Hypertension at baseline	16.6%	22.9%	18.1%	26.5%
Hypercholesterolemia at baseline	23.6%	30.8%	27.1%	44.4%
Hypertriglyceridemia at baseline	9.7%	15.7%	10.1%	14.6%

In the PREDIMED trial, during the active intervention period (2003–10; median follow-up: 4.8 years), 288 incident CVD cases were adjudicated by the Clinical Endpoint Committee and 357 deaths were confirmed.^1,26^ Over the extended follow-up period (mean follow-up: 13.4 years), 1930 deaths were documented. In the SUN cohort, during a 22-year total follow-up, 324 incident CVD events and 710 deaths were recorded.

Greater cumulative average compliance with the MedDiet was associated with lower risk of major CVD events and lower all-cause mortality in the PREDIMED study (*Table 3*; Supplementary data online, *Tables S1* and *S2*) and with a non-significant trend to lower all-cause mortality, but not CVD events, in the SUN cohort (*Table 3*; Supplementary data online, *Tables S3* and *S4*).

**Table 3:** Multivariable-adjusted hazard ratios for clinical outcomes and all-cause mortality in the PREDIMED trial (across different follow-up periods) and in the SUN cohort (restricted to participants older than 40 years), according to joint categories of the Mediterranean Diet Adherence Screener score and attainment of the wine point, using repeated measurements (cumulative averages) of both exposures

Wine point	MEDAS			
	Low MedDiet compliance		High MedDiet compliance	
	No wine	Adding wine	No wine	Adding wine
Cardiovascular disease				
PREDIMED (during the trial, 4.8 years follow-up)				
Number of cardiovascular events	113	59	80	36
MV1-adjusted HR (95% CI)	1 (ref.)	0.85 (0.60–1.21)	0.84 (0.61–1.15)	0.55 (0.36–0.83)
*P* value (wine vs no wine point)	.373		.067	
SUN participants >40 years (22 years follow-up)				
Number of cardiovascular events	215	73	28	8
MV2-adjusted HR (95% CI)	1.00 (ref.)	1.07 (0.81–1.42)	0.77 (0.49–1.22)	1.45 (0.74–2.83)
*P* value (wine vs no wine point)	.643		.099	
Pooled (PREDIMED 17 years + SUN)				
MV-adjusted HR (95% CI)	1.00 (ref.)	0.98 (0.79–1.22)	0.82 (0.63–1.06)	0.72 (0.51–1.03)
*P* value (wine vs no wine point)	.843		.317^a^	
All-cause mortality				
PREDIMED (during the trial, 4.8 years follow-up)				
Number of deaths	139	78	93	47
MV1-adjusted HR (95% CI)	1 (ref.)	0.92 (0.67–1.26)	0.67 (0.50–0.90)	0.48 (0.33–0.70)
*P* value (wine vs no wine point)	.589		.116	
PREDIMED (expanded 17 years follow-up)				
Number of deaths	680	303	646	301
MV1-adjusted HR (95% CI)	1 (ref.)	0.95 (0.82–1.10)	0.77 (0.68–0.87)	0.67 (0.57–0.78)
*P* value (wine vs no wine point)	.493		.021	
SUN participants >40 years (22 years follow-up)				
Number of deaths	478	151	73	8
MV2-adjusted HR (95% CI)	1.00 (ref.)	1.11 (0.91–1.35)	0.94 (0.71–1.26)	0.54 (0.28–1.04)
*P* value (wine vs no wine point)	.291		.067	
Pooled (PREDIMED 17 years + SUN)				
MV-adjusted HR (95% CI)	1.00 (ref.)	1.00 (0.89–1.13)	0.80 (0.71–0.89)	0.66 (0.57–0.77)
*P* value (wine vs no wine point)	.944		.010^b^	
Cardiovascular mortality				
PREDIMED (only fatal cases) (expanded 17 y follow-up)				
Number of cardiovascular deaths	189	83	143	77
MV1-adjusted HR (95% CI)	1.00 (ref.)	0.95 (0.71–1.27)	0.66 (0.51–0.84)	0.67 (0.49–0.92)
*P* value (wine vs no wine point)	.733		.713	
Cancer mortality				
PREDIMED (only fatal cases) (expanded 17 years follow-up)				
Cancer deaths	195	97	199	103
MV1-adjusted HR (95% CI)	1.00 (ref.)	0.86 (0.66–1.13)	0.80 (0.64–1.00)	0.65 (0.49–0.86)
*P* value (wine vs no wine point)	.278		.028	

MV1: multivariable model, with robust estimators of variance adjusted for age, smoking, diabetes, hypertension, dyslipidaemia, physical activity, waist-to-height ratio, body mass index (including a quadratic term), total energy intake, fruit consumption, vegetable consumption, and dietary fibre intake. All the models were stratified according to site, sex, educational level (five categories) and randomized arm of the trial. MV2: multivariable model with robust estimators of variance, adjusted for age (underlying time variable, and strata for decades), body mass index (adding a quadratic term), physical activity, years of university studies, smoking status, smoking pack-years, marital status, prevalence of depression, diabetes, hypertension, and cancer (also for prevalence of cardiovascular disease in models for total death), and consumption of other alcoholic beverages (excluding wine). Stratified by sex, year of entry to the cohort, and quartiles of total energy intake.

^a^The hazard ratios for cardiovascular disease when comparing wine vs no wine only within the stratum with good compliance with the MedDiet were 0.65 (0.40–1.03) in PREDIMED (4.8 years of median follow-up) and 2.56 (0.84–7.84) in the SUN cohort (up to 22 years follow-up). The pooled estimate was 0.80 (0.52–1.24), *P* = .317, with sufficient evidence of heterogeneity (*I*^2^ = 0.80, *Q* = 4.91, *P* = .03).

^b^The hazard ratios for total mortality when comparing wine vs no wine only within the stratum with good adherence to the MedDiet were 0.83 (0.70–0.97) in PREDIMED (up to 17 years follow-up) and 0.48 (0.22–1.05) in the SUN cohort (up to 22 years follow-up). The pooled estimate was 0.81 (0.69–0.95), *P* = .010, without only marginal heterogeneity (*I*^2^ = 0.45, *Q* = 1.81, *P* = .18).

**Figure 1:**
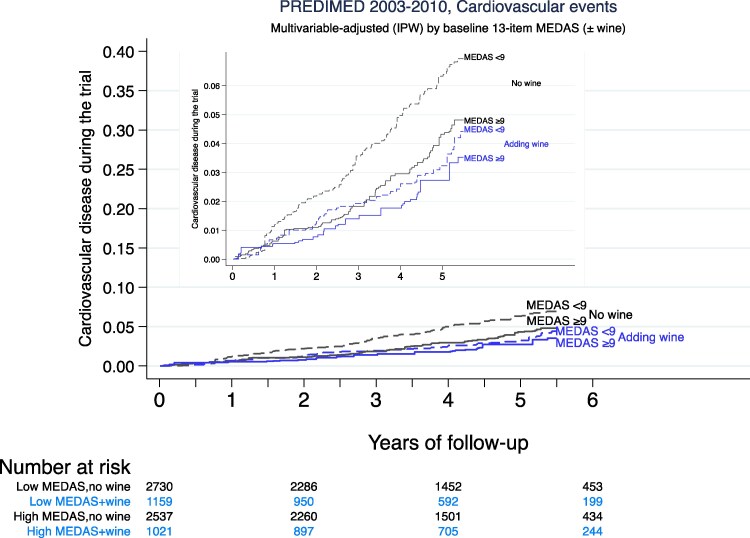
Inverse-probability weight-adjusted Nelson–Aalen curves for the primary endpoint of the PREDIMED trial (myocardial infarction, stroke, or cardiovascular death) by joint classification of adherence to the Mediterranean diet with or without the wine components of the Mediterranean Diet Adherence Screener during the intervention period (2003–10). Estimates were adjusted for age, sex, smoking, diabetes, hypertension, dyslipidaemia, physical activity, waist-to-height ratio, body mass index, total energy intake, fruit consumption, vegetable consumption, and dietary fibre intake

When analysing the primary CVD endpoint in the PREDIMED trial, the only group showing a significant association with lower risk than the reference group (low compliance with MedDiet and no wine) was the one with high compliance with the MedDiet and attainment of the wine point (*Table 3*; Supplementary data online, *Table S2*). When the analysis was restricted to the subset of participants who were good compliers with the MedDiet, the comparison between wine consumers and non-consumers yielded an HR of 0.65 (95% CI 0.40–1.03; *P* = .065) for CVD (see Supplementary data online, *Table S2*), suggesting an inverse association, though not statistically significant. However, among men, this inverse association reached statistical significance: restricting the analysis to good compliers with the MedDiet, men attaining the wine point exhibited a 45% lower risk as compared with good compliers without the wine point (HR 0.55; 95% CI 0.33–0.93; *P* = .025) (see Supplementary data online, *Table S2*). Overall, no significant supra-multiplicative interaction was found between MedDiet compliance and the wine point in a likelihood ratio test assessing this product-term (*P* = .32, 1 df). However, the linear trend for ordered categories of both combined exposures was significant (*P* = .007) (see Supplementary data online, *Table S5*). During the trial active intervention period, the inclusion of the baseline wine point was associated with lower CVD incidence across both categories of baseline MedDiet adherence (*Figure 1*).

**Figure 2:**
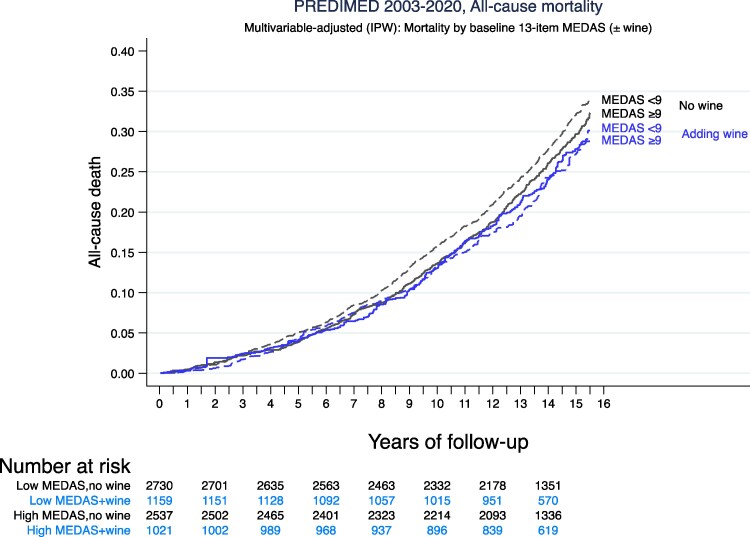
Inverse-probability weight-adjusted Nelson–Aalen curves for all-cause mortality by the joint classification of adherence to the Mediterranean diet with or without the inclusion of the wine component of the Mediterranean Diet Adherence Screener, during the extended follow-up period (2010–20). Estimates were adjusted using inverse-probability weighting for age, sex, smoking, diabetes, hypertension, dyslipidaemia, physical activity, waist-to-height ratio, body mass index (adding a quadratic term), total energy intake, fruit consumption, vegetable consumption, and dietary fibre intake

Regarding all-cause mortality during the extended follow-up period of PREDIMED, participants with low compliance with the MedDiet (MEDAS score ≤ 9) who, nevertheless, consumed ≥7 glasses/week of wine showed a non-significant inverse association with mortality risk (multivariable-adjusted HR 0.95; 95% CI 0.82–1.10), compared with those with low compliance and who did not consume wine (*Table 3*; Supplementary data online, *Table S2*). This association with lower mortality became more apparent and statistically significant among participants with higher MedDiet compliance (MEDAS > 9), but not reaching the wine threshold (<7 glasses/week), showing an association with 23% relatively lower mortality (HR 0.77; 95% CI 0.68–0.87). However, the association with the lowest all-cause mortality risk was found in participants who met both criteria (MEDAS > 9 and ≥7 glasses/week of wine), with an HR of 0.67 (95% CI 0.57–0.78; *P* = .02) (*Table 3*). When the analysis was restricted to only good compliers with the MedDiet (MEDAS > 9), wine consumers showed an association with 17% lower risk of mortality as compared with those who did not (HR 0.83; 95% CI 0.70–0.97; *P* = .021) (*Table 3*; Supplementary data online, *Table S2*), but the likelihood ratio test for supra-multiplicative interaction was not significant (*P* = .38, 1 df). The linear trend for ordered categories of combined exposures was highly significant (*P* < .001) (see Supplementary data online, *Table S5*). Thus, in the context of long-term mortality during the extended follow-up period, these findings suggest that including the wine component to a high compliance with the MedDiet was associated with a significantly lower all-cause mortality (*Figure 2*). Furthermore, during the extended follow-up of the PREDIMED study, greater MedDiet compliance was associated with lower mortality from both CVD and cancer causes. Interestingly, attainment of the wine point was significantly associated with lower cancer mortality (*P* = .03), but not with CVD mortality beyond the MedDiet effect (*Table 3*).

When we further adjusted for total alcohol intake in PREDIMED, the HR for incident CVD during the trial (comparing MedDiet + wine point vs neither) was 0.58 (95% CI 0.37–0.90). After removing excessive drinkers (>50 g/day), this HR was 0.54 (95% CI 0.35–0.83) (results not shown). For total mortality during the trial, the HR was 0.49 (95% CI 0.33–0.73) after adjusting for alcohol and 0.46 (95% CI 0.31–0.68) after removing excessive drinkers (results not shown). Various degrees of adjustment, including different sets of confounders, all yielded statistically significant linear trends, indicating associations with lower risk of CVD and with lower mortality with successive levels of increasing compliance with the MedDiet and including the wine component of the MEDAS score (see Supplementary data online, *Table S5*).

In the SUN cohort, no statistically significant associations were observed for CVD outcomes (*Table 3*). A non-significant trend to inverse association with mortality was observed in the SUN cohort among participants with both high MedDiet compliance and attainment of the wine point (*Table 3*; Supplementary data online, *Tables S3* and *S4*). Specifically, adding the wine point to high MedDiet compliance appeared associated with a non-significantly lower mortality (HR 0.48; 95% CI 0.22–1.05). Although this difference did not reach statistical significance in this younger and healthier SUN cohort (*Table 3*; Supplementary data online, *Table S4*), the direction of the association was consistent with findings from the PREDIMED study. Notably, the multiplicative interaction between MedDiet compliance and the wine point in relation to all-cause mortality was also non-significant (*P* = .08) in the SUN cohort. The multivariable-adjusted HRs for mortality in the SUN cohort, when using light alcohol intake (>0 and <5 g/day in women or >0 and <10 in men) as reference, were HR 1.18 (95% CI 0.94–1.49) for abstention; HR 1.27 (95% CI 1.07–1.52) for moderate intake (5–25 g/day for women and 10–50 g/day for men); and 1.85 (95% CI 1.18–2.90) for heavy alcohol intake (>25 g/day in women or >50 g/day in men). The respective multivariable-adjusted HRs for CVD were 0.90 (95% CI 0.62–1.30) for abstention; 1.12 (95% CI 0.87–1.45) for moderate intake; and 0.93 (95% CI 0.37–2.34) for heavy alcohol intake as compared with light intake (see Supplementary data online, *Table S4*).

*Table 4* shows the associations of total alcohol intake and total wine consumption with all-cause mortality in the extended follow-up of the PREDIMED trial, based on yearly cumulative averages derived from the FFQs. To reduce bias, diverse sensitivity analyses were performed, e.g. using the residual method, restricting the analysis to non-smokers, excluding abstainers, excluding former drinkers, or excluding those who reduced their ethanol intake during follow-up to minimize potential bias derived from the “sick quitter” effect. Additional sensitivity analyses changed the reference categories. Across all models, an approximate L-shaped association between alcohol intake (or wine consumption) and mortality risk was observed (*Table 4*). However, several of these analyses yielded non-significant results. Notably, no significant inverse associations with alcohol intake were found for total mortality when the reference category was changed to >0 and up to 2 drinks/week instead of using the abstainers as the reference group. The only significant finding was a higher risk in abstainers. Similar results were obtained for wine consumption (*Table 4*). After removing former drinkers, the multivariable-adjusted HRs were 0.81 (95% CI 0.70–0.93) for 0.5–<2 drinks/day of total ethanol and 0.85 (95% CI 0.70–1.03) for two or more drinks/day (results not shown). For wine consumption, these estimates were 0.79 (95% CI 0.69–0.91) for 0.5–<2 drinks/day, and 0.90 (95% CI 0.74–1.10) for 2 or more drinks/day (results not shown).

**Table 4:** Multivariable-adjusted hazard ratios for all-cause mortality during the extended follow-up of the PREDIMED trial (2003–20), according to cumulative exposures to total alcohol intake and to wine consumption

Long-term mortality in the PREDIMED trial (up to 17 years of follow-up)					
	Cumulative average of alcohol intake (drinks/week or drinks/day) during each year of the trial				
	Abstention (0)	>0–<1/week	1/week–0.5/day	0.5/day–<3/day	≥3/day
Total	1730	1429	1283	2570	383
Person-years	24 723	17 948	16 896	34 473	5384
Deaths	520	346	276	662	109
Age-, sex-adjusted HR (95% CI)	1 (ref.)	0.86 (0.75–0.99)	0.73 (0.62–0.85)	0.77 (0.67–0.87)	0.84 (0.67–1.05)
MV-adjusted HR (95% CI)	1 (ref.)	0.88 (0.77–1.02)	0.77 (0.66–0.90)	0.80 (0.70–0.92)	0.80 (0.63–1.03)
MV-adjusted HR (95% CI) and removing former drinkers	1 (ref.)	0.89 (0.77–1.03)	0.76 (0.65–0.90)	0.82 (0.71–0.94)	0.82 (0.64–1.05)
MV-adjusted (residual^a^) HR (95% CI)	1 (ref.)	0.86 (0.72–1.01)	0.82 (0.69–0.97)	0.82 (0.70–0.96)	0.85 (0.66–1.09)
Restricting the analysis to non-smokers (2912 participants excluded)					
Age-, sex-adjusted HR (95% CI)	1 (ref.)	0.88 (0.75–1.03)	0.79 (0.66–0.95)	0.76 (0.64–0.91)	0.80 (0.46–1.40)
MV-adjusted HR (95% CI)	1 (ref.)	0.89 (0.75–1.05)	0.83 (0.68–1.01)	0.84 (0.70–1.02)	0.97 (0.56–1.68)
Removing initial drinkers who quitted alcohol during follow-up (283 participants excluded)					
Age-, sex-adjusted HR (95% CI)	1 (ref.)	0.82 (0.71–0.96)	0.71 (0.61–0.83)	0.76 (0.67–0.87)	0.83 (0.66–1.04)
MV-adjusted HR (95% CI)	1 (ref.)	0.84 (0.73–0.98)	0.75 (0.64–0.88)	0.80 (0.70–0.91)	0.80 (0.63–1.02)
Removing also drinkers who reduced intake to <5 g/day during follow-up (472 participants excluded)^b^					
Age-, sex-adjusted HR (95% CI)	1 (ref.)	0.86 (0.75–0.99)	0.72 (0.61–0.85)	0.77 (0.67–0.87)	0.82 (0.65–1.03)
MV-adjusted HR (95% CI)	1 (ref.)	0.89 (0.77–1.02)	0.77 (0.65–0.91)	0.81 (0.70–0.93)	0.79 (0.62–1.02)
Removing ALL abstainers (1730 participants excluded)		>0–<1/week	1/week–0.5/day	0.5/day–<2/day	≥2/day
Age-, sex-adjusted HR (95% CI)		1 (ref.)	0.85 (0.72–0.99)	0.88 (0.76–1.01)	0.97 (0.81–1.16)
MV-adjusted HR (95% CI)		1 (ref.)	0.88 (0.75–1.04)	0.91 (0.78–1.05)	0.97 (0.80–1.18)
>0 up to 2 drinks/week as reference	Abstention (0)	>0–≤2/week	>2/week–0.5/day	0.5/day–<2/day	≥2/day
Age-, sex-adjusted HR (95% CI) and removing former drinkers	1.24 (1.09–1.41)	1 (ref.)	0.97 (0.80–1.18)	0.95 (0.83–1.09)	1.03 (0.87–1.23)
MV-adjusted HR (95% CI) and removing former drinkers	1.21 (1.06–1.39)	1 (ref.)	1.04 (0.86–1.26)	0.99 (0.86–1.13)	1.04 (0.87–1.25)
Baseline intake as exposure and >0 up to 2 drinks/week as reference	Abstention (0)	>0–≤2/week	>2/week–0.5/day	0.5/day–<2/day	≥2/day
Age-, sex-adjusted HR (95% CI) and removing former drinkers	1.09 (0.95–1.25)	1 (ref.)	1.09 (0.88–1.35)	0.90 (0.77–1.05)	1.05 (0.89–1.25)
MV-adjusted HR (95% CI) and removing former drinkers	1.02 (0.88–1.17)	1 (ref.)	1.10 (0.89–1.37)	0.91 (0.78–1.07)	1.00 (0.84–1.20)

	Cumulative average of wine consumption (drinks/week or drinks/day) during each year of the trial				
	Abstention (0)	>0–<1/week	1/week–0.5/day	0.5/day–<3/day	≥3/day
Total	2373	1206	1471	2190	155
Person-years	33 578	14 886	19 619	29 111	2230
Deaths	687	291	315	575	45
Age-, sex-adjusted HR (95% CI)	1 (ref.)	0.89 (0.78–1.03)	0.76 (0.66–0.87)	0.77 (0.68–0.87)	0.81 (0.59–1.11)
MV-adjusted HR (95% CI)	1 (ref.)	0.92 (0.80–1.07)	0.80 (0.69–0.92)	0.79 (0.70–0.91)	0.81 (0.57–1.13)
MV-adjusted HR (95% CI) and removing former drinkers	1 (ref.)	0.94 (0.81–1.08)	0.81 (0.70–0.94)	0.81 (0.71–0.93)	0.85 (0.60–1.19)
MV-adjusted (residual^a^) HR (95% CI)	1 (ref.)	0.85 (0.72–0.99)	0.79 (0.67–0.93)	0.79 (0.67–0.92)	0.81 (0.57–1.16)
Restricting the analysis to non-smokers					
Age-, sex-adjusted HR (95% CI)	1 (ref.)	0.92 (0.78–1.09)	0.80 (0.67–0.96)	0.81 (0.68–0.97)	0.49 (0.20–1.19)
MV-adjusted HR (95% CI)	1 (ref.)	0.91 (0.77–1.09)	0.83 (0.68–1.00)	0.89 (0.74–1.08)	0.58 (0.24–1.41)
Removing initial drinkers who quitted alcohol during follow-up					
Age-, sex-adjusted HR (95% CI)	1 (ref.)	0.84 (0.73–0.98)	0.74 (0.64–0.86)	0.76 (0.67–0.86)	0.81 (0.59–1.11)
MV-adjusted HR (95% CI)	1 (ref.)	0.86 (0.74–1.00)	0.78 (0.68–0.90)	0.78 (0.69–0.90)	0.80 (0.57–1.13)
Removing also drinkers who reduced intake to <5 g/day during follow-up^b^					
Age-, sex-adjusted HR (95% CI)	1 (ref.)	0.89 (0.77–1.03)	0.75 (0.64–0.87)	0.77 (0.68–0.87)	0.80 (0.58–1.10)
MV-adjusted HR (95% CI)	1 (ref.)	0.93 (0.80–1.07)	0.78 (0.67–0.91)	0.80 (0.70–0.91)	0.82 (0.58–1.15)
Removing ALL abstainers				0.5/day–<2/day	≥2/day
Age-, sex-adjusted HR (95% CI)		1 (ref.)	0.86 (0.73–1.01)	0.82 (0.70–0.96)	0.99 (0.81–1.22)
MV-adjusted HR (95% CI)		1 (ref.)	0.87 (0.73–1.03)	0.84 (0.71–0.99)	0.97 (0.78–1.21)
>0 up to 2 glasses/week as reference	Abstention (0)	>0–≤2/week	>2/week–0.5/day	0.5/day–<2/day	≥2/day
Age-, sex-adjusted HR (95% CI)	1.24 (1.10-1.40)	1 (ref.)	1.08 (0.89–1.30)	0.92 (0.80–1.05)	1.10 (0.92–1.33)
MV-adjusted HR (95% CI)	1.20 (1.06-1.36)	1 (ref.)	1.12 (0.92–1.35)	0.93 (0.81–1.07)	1.08 (0.89–1.32)
Baseline intake as exposure and >0 up to 2 glasses/week as reference	Abstention (0)	>0–≤2/week	>2/week–0.5/day	0.5/day–<2/day	≥2/day
Age-, sex-adjusted HR (95% CI)	1.13 (0.99–1.30)	1 (ref.)	0.98 (0.80–1.19)	0.85 (0.72–1.01)	1.12 (0.94–1.34)
MV-adjusted HR (95% CI)	1.08 (0.94–1.24)	1 (ref.)	0.99 (0.81–1.20)	0.87 (0.73–1.03)	1.06 (0.88–1.29)

Mortality outcomes were assessed over up to 17 years of follow-up.

MV: multivariable, adjusted for age, smoking, diabetes, hypertension, dyslipidaemia, physical activity, waist-to-height ratio, body mass index (including a quadratic term), total energy intake, fruit consumption, vegetable consumption, and dietary fibre intake, a robust variance estimator was used and the models were stratified according to site, sex, educational level (five categories), and randomized arm of the trial.

^a^The residual method was used to adjust alcohol intake (g/day) or wine consumption (mL/day) for total energy intake, separately for men and women.

^b^All participants who were drinkers at baseline and quitted alcohol during follow-up (*n* = 283) were removed and also those who drank at baseline > 5 g/day of alcohol, but subsequently reduced their average intake to ≤5 g/day during follow-up (*n* = 189). In total, 472 participants were excluded in this sensitivity analysis.

**Figure 3:**
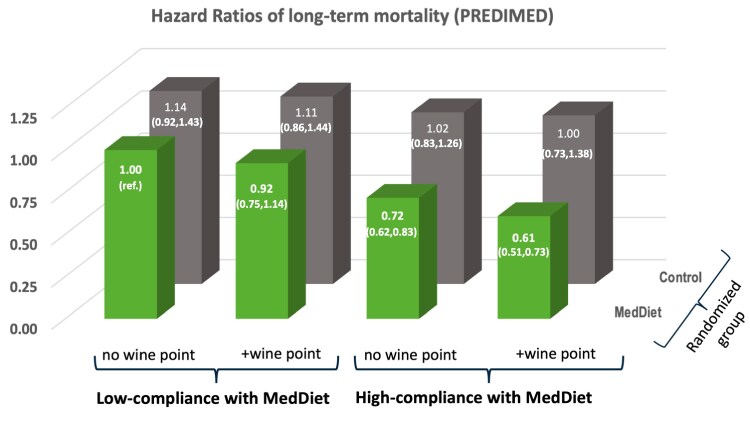
Relative risks of all-cause mortality in the PREDIMED trial according to the combination of three dichotomous exposures: (i) random allocation in the trial (merging the two Mediterranean diet groups vs the control group); (ii) actual dietary compliance with the Mediterranean diet according to the Mediterranean Diet Adherence Screener (0–13 points, after excluding wine): low compliance, ≤9 points vs high compliance, >9 points; and (iii) attainment or not of the wine item of the intervention (≥7 glasses/week)

In the PREDIMED study, when assessing the combination of three dichotomous exposures (randomly allocated arm, adherence to the MedDiet, and attainment of the wine point on the MEDAS score), an association with lower long-term mortality was found among participants randomly allocated to either of the two MedDiet intervention groups. Moreover, better compliance with the MedDiet was associated with lower mortality. Importantly, the addition of the wine point was associated with the numerically lowest risk of all-cause mortality (*Figure 3*). However, most of these associations showed overlapping CIs, given the sparse data in most categories (see Supplementary data online, *Table S6*).

Supplementary data online, *Table S7* shows results of PREDIMED under a classification of alcohol intake (or wine) recently used by other investigators^32^ who restricted their analysis to participants ≥60 years. We also restricted this analysis to participants 60 years and older, excluded former drinkers and abstainers, and used as reference category an ethanol intake of >0–≤2.86 g/day, as they did, the multivariable-adjusted HR was 1.02 (95% CI 0.85–1.22) for moderate drinkers using this reference. In the SUN cohort, using this same approach, we found the lowest risk for occasional and light drinkers (see Supplementary data online, *Table S7*). Supplementary data online, *Table S8* shows another alternative classification merging several categories. Non-significant results were found in this sensitivity analysis. In an evaluation of baseline prevalence in PREDIMED of a wide array of major diseases by drinking habits (see Supplementary data online, *Table S9*), the only disease which was significantly more frequent at baseline in former drinkers than in current drinkers was depression. But additional adjustment for depression did not change our results.

In the pooled analysis of both the PREDIMED and SUN cohorts, using low MedDiet compliance and no wine as the reference category, the combination of high MedDiet compliance and wine consumption was not associated with lower CVD risk (HR 0.72; 95% CI 0.51–1.03) but was associated with lower all-cause mortality (HR 0.66; 95% CI 0.57–0.77) (*Table 3*). Furthermore, in this pooled analysis of both cohorts, within the subset of good MedDiet compliers, there was a statistically significant difference in mortality risk between wine and no wine consumers (*P* = .01) (*Table 3*).

## Discussion

In an extended follow-up of the PREDIMED trial, inverse associations were independently observed between compliance with the MedDiet, including moderate wine consumption, and the risk of either CVD or all-cause mortality. Notwithstanding, it should be acknowledged that the multiplicative interaction between adherence to the MedDiet and the wine point did not reach statistical significance and several sensitivity analyses were non-significant. Also, in the younger and healthier SUN cohort, no significant differences were found for CVD (with point estimates even trending in the opposite direction) and a higher risk of all-cause mortality was observed for moderate drinkers as compared with light alcohol intake. Nevertheless, the pooled estimates of both cohorts resulted in a non-significant trend to lower risk of CVD and a significant association with lower all-cause mortality when the wine item was added to the MedDiet. These inverse associations between moderate wine consumption and lower all-cause mortality remained consistent across both cohorts among participants with high adherence to the MedDiet (*Structured Graphical Abstract*).

Notably, in the PREDIMED trial, both random allocation to the MedDiet intervention and actually attained compliance with the MedDiet were independently associated with lower mortality. Furthermore, adding the wine point of the MEDAS score was associated with the lowest numerical HR for all-cause mortality, as compared with the respective reference categories (*Figure 3*; Supplementary data online, *Table S6*). It is important to emphasize that these analyses are observational in nature, as wine consumption was not independently randomized in either of the cohorts studied. Still, moderate wine consumption was a recommended component of the randomized dietary intervention, as advised by dietitians to participants in the two MedDiet arms of the PREDIMED trial, provided they were already drinkers at baseline. Notwithstanding, various alternative reasons might explain our findings. In fact, wine drinkers could be assumed to be systematically different from non-wine drinkers. In PREDIMED, they were more physically active and had higher education; however, they were also more likely to be smokers and all these factors were controlled for in the analyses. More importantly, a simple comparison of less than one drink of wine per day vs at least one may suffer from various kinds of reference group bias. In fact, when we alternatively analysed total ethanol intake using occasional or light drinkers as the reference category, some estimates showed higher risks among moderate drinkers in the SUN cohort (see Supplementary data online, *Tables S4* and *S7*).

For decades, a substantial body of prospective epidemiologic evidence has supported an inverse association between low-to-moderate wine consumption and all-cause mortality.^3,10,12,15–17,20,24,33^ Similarly, a non-linear association between wine consumption and CVD incidence, including fatal and non-fatal cases, has been consistently reported across multiple studies.^16,19,20,24,33^

In the context of an ongoing and intense debate regarding the potential health effects of alcohol, the US National Academies concluded in 2025 with moderate certainty that “compared with never consuming alcohol, moderate alcohol consumption is associated with lower all-cause mortality”.^34^ They defined moderate alcohol consumption as two drinks per day (28 g of alcohol) for men and one drink per day (14 g of alcohol) for women. However, this conclusion might need to be toned down, given that the same Academies recommend that “individuals should not start drinking for any reason and that drinking less is better for health than drinking more”. In addition, the 2025 recommendations by the US Surgeon General^6^ advocated universal alcohol abstention in the interest of cancer prevention. These recommendations were issued almost simultaneously (January 2025), reflecting some degree of ongoing divergent interpretations of the available evidence. Also, the National Academies’ reliance on all-cause mortality as primary endpoint was criticized, as approximately 95% of deaths are unrelated to alcohol, raising concerns for potential misclassification and reverse causation in such analyses.^35,36^

The Global Burden of Disease (GBD) 2016 study^37^ reported that “no level of alcohol consumption improves health”.^38^ However, the GBD 2020 update, 4 years later, introduced a more nuanced perspective based on a novel systematic review and meta-analysis. It defined theoretical minimum risk exposure levels above 0 (personalized by world region, age, and sex) and reported that “small amounts of alcohol consumption are associated with improved health outcomes in populations facing a high burden of cardiovascular diseases”.^39^ Despite these revised estimates, concerns persist regarding the biological plausibility of these findings and the elevated values proposed as non-drinker equivalents, particularly for older populations, which may warrant further scrutiny.^40,41^

A common conclusion across recent reports is the urgent need for further investigation, ideally through randomized controlled trials, as recently recommended by the American Heart Association.^41^ In this context, the present observational analyses with yearly repeated measurements of exposure and an extended mortality follow-up (2003–20) of the PREDIMED trial are novel. Specifically, they provide evidence from a trial of the MedDiet, in which moderate wine consumption was a recommended component of the randomized intervention. Beyond the overall effect of the MedDiet, daily moderate wine consumption was independently associated with lower all-cause mortality, and the wine component of the MEDAS score was additionally associated with lower total CVD risk during the active intervention period of the trial (2003–10). These findings align with those from two large British cohorts, namely, the CALIBER study^42^ and the UK Biobank,^33^ where, among 354 386 current drinkers, a significant U-shaped relationship was found between wine consumption and both all-cause and non-cancer mortality. However, lack of representativeness of the UK Biobank may contribute to underestimate the true risks associated with alcohol.^43^ In any case, the lowest all-cause mortality was observed at 19–23 g/day of alcohol intake, primarily from wine. In contrast, non-wine alcohol intake was associated with higher mortality.^33^ Similarly, in the PREDIMED analysis, a comparable relationship was also observed between total alcohol intake (mainly from wine) and long-term mortality, based on repeated yearly FFQs. This pattern persisted even after adjusting for the “sick quitter” effect. Notably, PREDIMED explicitly excluded individuals with alcohol use disorders or former alcoholics, minimizing potential biases due to reverse causation. When testing whether abstainers or former drinkers were unhealthier at baseline (the main assumption underlying these potential biases), PREDIMED data showed that, with the exception of depression (more prevalent in abstainers), no other previous disease was significantly more prevalent in abstainers or former drinkers as compared with light or moderate drinkers. But sensitivity analyses suggested that no confounding was exerted by prevalent depression, thus allaying concerns of potential “sick quitter” biases. Nevertheless, we acknowledge that these supposed biases cannot be completely ruled out in our study, given our observational approach and the inherent limitations of our measurement tools.

Stockwell *et al*.^44^ reported that previous studies presenting results specifically for non-smokers did not find lower mortality for low-volume drinkers and suggested that adjustment for smoking may contribute to generate apparent J-shaped relationships between alcohol intake and mortality. In this context, sensitivity analyses restricted to non-smokers are informative because they pre-empt confounding by smoking. However, these results restricted to non-smokers did not substantially diverge from the main analysis in PREDIMED, with CIs including the point estimates from the full sample analysis and the point estimates for moderate drinkers changed <10%.

Several biological mechanisms may explain the associations between low-to-moderate alcoholic beverage intake, specifically wine consumption, and lower mortality rates. These include anti-inflammatory^45^ and antioxidant properties, increases in HDL cholesterol (although whether high HDL levels will protect against coronary heart disease is now debated^46^), reduction of oxidized LDL cholesterol, improvements in endothelial function, enhanced insulin sensitivity, reduced platelet aggregation, changes in metabolic pathways and plasma metabolites,^5^ and modulation of the gut microbiome.^18^ Some of these effects may be attributed to the ethanol content, while many others are attributed to various (poly)phenolic compounds mainly present in wine.^47,48^ Our main focus was wine, not alcohol intake.

In this context, low-to-moderate wine consumption in the PREDIMED study was associated with up to 50% relatively lower major cardiovascular events in a subset of participants, when objective biomarkers of wine consumption, such as urinary tartaric acid concentrations, were used.^48^ Our findings should also be interpreted in the context of recent literature considering self-reported total alcohol intake, instead of assessing only wine, which produced alternative results for total alcohol intake. These studies did not replicate J-shaped curves previously described, whether for all-cause mortality or for other chronic diseases in relationship with alcohol intake.^32^

In addition to examining the association of wine in the context of a MedDiet intervention with CVD and mortality in PREDIMED, another independent Spanish cohort was assessed: the SUN cohort with 22 years of follow-up and including younger and healthier participants. Results were not statistically significant, but they showed a similar direction of the association of the wine item with all-cause mortality (though not for CVD). A limitation to be acknowledged is that the exposure was updated only 10 years apart in SUN, but annually updated in PREDIMED. Notwithstanding, the pooled analysis of both cohorts revealed lower risk for death when wine consumption was included as part of the MedDiet.

Despite the diverse and extensive adjustments for potential confounders, the possibility of residual confounding cannot be fully excluded in these two cohorts, as it is inherent in all observational studies. Income data were not available. However, to further minimize the risk of residual confounding by social status, we applied education-level restrictions in the SUN cohort and, in the PREDIMED trial, we adjusted for educational level through stratification in the risks sets. Differences between the two cohorts in the observed associations of moderate wine consumption may be attributable to variations in age and underlying CVD burden.^39^ In the SUN cohort, the lower number of deaths and the fewer participants who met both criteria (high MEDAS compliance and wine consumption) among those aged 40 years or older made it infeasible to restrict analyses to older subgroups. However, in both cohorts, it needs to be acknowledged that interactions did not reach statistical significance and that some associations showed considerable uncertainty; therefore, results should be interpreted with caution. Particularly, when the reference category was changed to drinkers of >0 and up to 2 drinks/week, only abstainers showed an association with an increased mortality in PREDIMED, but the results for upper levels of both alcohol and wine consumption were non-significant. Additionally, full replication across cohorts was not achieved, which may limit the generalizability of our findings.

In conclusion, over a long-term follow-up of individuals at high cardiovascular risk in the PREDIMED trial, moderate wine consumption, as part of the MedDiet intervention, was associated with lower risks for both total CVD incidence and all-cause mortality. These findings were not replicated in a younger cohort (SUN). In addition, exploratory dose–response analyses did not demonstrate reduced risk for death in PREDIMED participants who drank three or more glasses of wine/day (suggesting the favourable associations only apply to low-to-moderate consumption and not high wine consumption). To confirm these observations, large-scale randomized controlled trials, specifically designed to compare moderate wine consumption vs abstention wine, are warranted.

## Supplementary Material

ehaf1081_Supplementary_Data

## Declarations

### Data Availability

The datasets generated and/or analysed during the current study are available from the corresponding author upon reasonable request. Due to privacy or ethical restrictions, individual-level data are not publicly available.

### Pre-registered Clinical Trial Number

This trial was registered in Current Controlled Trials, London (ISRCTN357396693) funded by the Instituto de Salud Carlos III, Spanish Government.
