# Supplementary material for: Wine consumption, Mediterranean diet, and cardiovascular risk in two Spanish cohorts
Source: Eur Heart J. 2026 Feb 11;47(27):3591–606. doi: 10.1093/eurheartj/ehaf1081 (PMC13364079; doi:10.1093/eurheartj/ehaf1081)
Supplement: ehaf1081_Supplementary_Data [file ehaf1081_supplementary_data.zip › Supplementary Table 1.docx]

**Supplementary Table 1**. Hazard ratios (HRs) for all-cause mortality during the extended follow-up period of the PREDIMED trial (2003-2020), by compliance with the Mediterranean Diet (assessed by the Mediterranean Diet Adherence Screener, MEDAS) with or without the addition of the MEDAS point for consuming at least 1 glass/d of wine, using repeated cumulative averaged annual measurements, and including sensitivity analysis for total wine consumption and total alcohol intake derived from the yearly repeated food frequency questionnaires (FFQ).

| **ALL-CAUSE MORTALITY (up to 17 years follow-up)** | | | | | |
| --- | --- | --- | --- | --- | --- |
|  | **Cumulative average MEDAS with or without wine during follow-up** | | | |  |
| **MEDAS (without wine)** | **Low MedDiet compliance (0 to 9)** | | **High MedDiet compliance (>9 to 13)** | | **p value**  **(wine vs. no wine)** |
| **Wine point during follow-up** | **No wine** | **Adding wine/alcohol** | **No wine** | **Adding wine/alcohol** |  |
| **All (n)** | 2299 | 913 | 2942 | 1293 |  |
| Person-years | 32360 | 13554 | 37214 | 16961 |  |
| Deaths | 680 | 303 | 646 | 301 |  |
| Age-, sex-adjusted HR (95% CI) | 1 (ref.) | 0.98 (0.84 - 1.13)^a^ | 0.81 (0.73 - 0.91) | 0.70 (0.61 - 0.80)^b^ | ^a^p = 0.73 ^b^p = 0.06 |
| MV-adjusted HR (95% CI) | 1 (ref.) | 0.95 (0.82 - 1.10)^a^ | 0.77 (0.68 - 0.87) | 0.67 (0.57 - 0.78)^b^ | ^a^p = 0.49 ^b^p = 0.021 |
| MV-adjusted HR with FFQ (wine) | 1 (ref.) | 1.00 (0.86 - 1.16)^a^ | 0.78 (0.69 - 0.89) | 0.67 (0.57 - 0.79)^b^ | ^a^p = 0.98 ^b^p = 0.008 |
| MV-adjusted HR (alcohol 10+ g/d) | 1 (ref.) | 1.05 (0.90 - 1.21)^a^ | 0.80 (0.70 - 0.90) | 0.70 (0.59 - 0.82)^b^ | ^a^p = 0.56 ^b^p = 0.018 |
| *MV-adjusted HR (95% CI) only within good MedDiet compliers:* | | | *1 (ref.)* | *0.83 (0.70 - 0.97)* | *p = 0.021* |
| **Men (n)** | 728 | 657 | 868 | 912 |  |
| Person-years | 9683 | 9548 | 10420 | 11645 |  |
| Deaths | 268 | 234 | 242 | 242 |  |
| Age-adjusted HR (95% CI) | 1 (ref.) | 0.92 (0.77 - 1.10)^a^ | 0.77 (0.65 - 0.91) | 0.70 (0.59 - 0.83)^b^ | ^a^p = 0.36 ^b^p = 0.33 |
| MV-adjusted HR (95% CI) | 1 (ref.) | 0.88 (0.73 - 1.06)^a^ | 0.72 (0.59 - 0.87) | 0.65 (0.53 - 0.79)^b^ | ^a^p = 0.18 ^b^p = 0.15 |
| *MV-adjusted HR (95% CI) only within good MedDiet compliers:* | | | *1 (ref.)* | *0.87 (0.71 - 1.05)* | *p = 0.149* |
| **Women (n)** | 1571 | 256 | 2074 | 381 |  |
| Person-years | 22677 | 4005 | 26794 | 5317 |  |
| Deaths | 412 | 69 | 404 | 59 |  |
| Age-adjusted HR (95% CI) | 1 (ref.) | 1.09 (0.84 - 1.42)^a^ | 0.84 (0.73 - 0.97) | 0.64 (0.49 - 0.84)^b^ | ^a^p = 0.51 ^b^p = 0.06 |
| MV-adjusted HR (95% CI) | 1 (ref.) | 1.10 (0.84 - 1.43)^a^ | 0.82 (0.70 - 0.96) | 0.65 (0.49 - 0.87)^b^ | ^a^p = 0.49 ^b^p = 0.07 |
| *MV-adjusted HR (95% CI) only within good MedDiet compliers:* | | | *1 (ref.)* | *0.76 (0.57 - 1.02)* | *p = 0.065* |

^a^: versus low compliance and no wine; ^b^: versus *high* compliance and no wine (only p values). MV: multivariable, adjusted for age, smoking, diabetes, hypertension, dyslipidemia, physical activity, waist-to-height ratio, body mass index (including a quadratic term), total energy intake, fruit consumption, vegetable consumption, and dietary fiber intake, a robust variance estimator was used and the models were stratified according to site, sex, educational level (five categories) and randomized arm of the trial.
