# Supplementary material for: Wine consumption, Mediterranean diet, and cardiovascular risk in two Spanish cohorts
Source: Eur Heart J. 2026 Feb 11;47(27):3591–606. doi: 10.1093/eurheartj/ehaf1081 (PMC13364079; doi:10.1093/eurheartj/ehaf1081)
Supplement: ehaf1081_Supplementary_Data [file ehaf1081_supplementary_data.zip › Supplementary Table 2.docx]

**Supplementary Table 2**. Hazard ratios (HRs) for cardiovascular events during the active intervention period of the PREDIMED trial (2003-2010), by cumulative averages of compliance with the Mediterranean Diet (repeatedly assessed by the Mediterranean Diet Adherence Screener, MEDAS) with or without the addition of the point for consuming at least 1 glass/d of wine.

| **CARDIOVASCULAR EVENTS DURING THE ACTIVE INTERVENTION IN PREDIMED TRIAL (4.8 years of follow-up)** | | | | |
| --- | --- | --- | --- | --- |
|  | **Cumulative average MEDAS with or without wine during follow-up** | | | |
| **MEDAS (without wine)** | **Low MedDiet compliance (0 to 9)** | | **High MedDiet compliance (>9 to 13)** | |
| **Wine point during follow-up** | **No wine** | **Adding wine** | **No wine** | **Adding wine** |
| **Total** | 2450 | 1036 | 2727 | 1234 |
| Person-years | 11422 | 5481 | 10010 | 5066 |
| Cases of CVD | 113 | 59 | 80 | 36 |
| Age-, sex-adjusted HR (95% CI) | 1 (ref.) | 0.83 (0.60 - 1.16) | 0.78 (0.59 - 1.04) | 0.51 (0.35 - 0.76) |
| MV-adjusted HR (95% CI) | 1 (ref.) | 0.85 (0.60 - 1.21) | 0.84 (0.61 - 1.15) | 0.55 (0.36 - 0.83) |
| *MV-adjusted HR (95% CI) for wine vs. no wine only among good MedDiet compliers:* | | | **1 (ref.)** | **0.65 (0.40 - 1.03)*** |
| **Men** |  |  |  |  |
| Cases of CVD | 54 | 49 | 37 | 28 |
| Age-adjusted HR (95% CI) | 1 (ref.) | 0.79 (0.54 - 1.15) | 0.74 (0.49 - 1.12) | 0.45 (0.29 - 0.71) |
| MV-adjusted HR (95% CI) | 1 (ref.) | 0.83 (0.55 - 1.25) | 0.78 (0.49 - 1.24) | 0.48 (0.30 - 0.77) |
| *MV-adjusted HR (95% CI) for wine vs. no wine only among good MedDiet compliers:* | | | **1 (ref.)** | **0.55 (0.33 - 0.93)**** |
| **Women** |  |  |  |  |
| Cases of CVD | 56 | 10 | 43 | 8 |
| Age-adjusted HR (95% CI) | 1 (ref.) | 0.90 (0.46 - 1.77) | 0.83 (0.56 - 1.25) | 0.74 (0.35 - 1.56) |
| MV-adjusted HR (95% CI) | 1 (ref.) | 0.79 (0.40 - 1.57) | 0.95 (0.61 - 1.48) | 0.85 (0.39 - 1.89) |
| *MV-adjusted HR (95% CI) for wine vs. no wine only among good MedDiet compliers:* | | | **1 (ref.)** | **0.99 (0.41 - 2.39)** |

MV: multivariable, adjusted for age, smoking, diabetes, hypertension, dyslipidemia, physical activity, waist-to-height ratio, body mass index (including a quadratic term), total energy intake, fruit consumption, vegetable consumption, and dietary fiber intake, a robust variance estimator was used and the models were stratified according to site, sex, educational level (five categories) and randomized arm of the trial.

* P = 0.065 in the likelihood ratio test. **P = 0.034 in the likelihood ratio test.
