# Supplementary material for: Wine consumption, Mediterranean diet, and cardiovascular risk in two Spanish cohorts
Source: Eur Heart J. 2026 Feb 11;47(27):3591–606. doi: 10.1093/eurheartj/ehaf1081 (PMC13364079; doi:10.1093/eurheartj/ehaf1081)
Supplement: ehaf1081_Supplementary_Data [file ehaf1081_supplementary_data.zip › Supplementary Table 3.docx]

**Supplementary Table 3**. Hazard ratios (HRs) for all-cause mortality in the SUN cohort among participants aged >40 years, according to joint categories of baseline MEDAS score (excluding the wine item) and baseline wine consumption.

| **ALL-CAUSE MORTALITY (up to 22 years follow-up)** | | | | |
| --- | --- | --- | --- | --- |
|  | **Baseline MEDAS with or without wine** | | | |
| **Baseline MEDAS (without wine)** | **Low MedDiet compliance (0 to 8)** | | **High MedDiet compliance (9 to 13)** | |
| **Wine point at baseline** | **No wine** | **Adding wine** | **No wine** | **Adding wine** |
| **All (n)** | 7890 | 1544 | 969 | 151 |
| Deaths | 484 | 161 | 57 | 8 |
| Person-years | 118,196 | 23,586 | 13,866 | 2,288 |
| Mortality rate 10^-3^ | 4.09 | 6.83 | 4.11 | 3.50 |
| Sex-Age-adjusted HR (95% CI) | 1.00 (Ref) | 1.16 (0.97 - 1.38) | 0.79 (0.60 - 1.04) | 0.49 (0.24 - 0.99) |
| MV-adjusted HR (95% CI) | 1.00 (Ref) | 1.15 (0.93 - 1.42) | 0.74 (0.52 - 1.07) | 0.51 (0.24 - 1.09) |
| *MV-adjusted HR (95% CI) for wine vs. no wine only among good MedDiet compliers:* | | | **1 (ref.)** | **0.68 (0.30 - 1.56)** |

MV: multivariable model with robust estimators of variance, adjusted for age (underlying time variable, and strata for decades), body mass index (adding a quadratic term), physical activity, years of university studies, smoking status, smoking pack-years, marital status, prevalence of depression, diabetes, hypertension, cancer and cardiovascular disease, consumption of other alcoholic beverages (excluding wine). Stratified by sex, year of entry to the cohort and quartiles of total energy intake.
