# Supplementary material for: Wine consumption, Mediterranean diet, and cardiovascular risk in two Spanish cohorts
Source: Eur Heart J. 2026 Feb 11;47(27):3591–606. doi: 10.1093/eurheartj/ehaf1081 (PMC13364079; doi:10.1093/eurheartj/ehaf1081)
Supplement: ehaf1081_Supplementary_Data [file ehaf1081_supplementary_data.zip › Supplementary table 4.docx]

**Supplementary Table 4**. Hazard ratios (HRs) for all-cause mortality and cardiovascular disease in SUN cohort participants aged >40 years, by joint categories of the MEDAS score and wine consumption, and according to ethanol intake, using repeated measurements of intake (cumulative averages of baseline and 10-year follow-up).

| **ALL-CAUSE MORTALITY (up to 22 years follow-up)** | | | | | | | | | |
| --- | --- | --- | --- | --- | --- | --- | --- | --- | --- |
|  | | **Cumulative average of MEDAS with or without wine** | | | | | |  | |
| **Cumulative average of MEDAS (without wine)** | | **Low MedDiet compliance**  **(0 to 8)** | | | **High MedDiet compliance**  **(9 to 13)** | | | **p value**  **(wine vs. no wine)** | |
| **Wine point at baseline** | | **No wine** | **Adding wine** | | **No wine** | **Adding wine** | |  | |
| **n** | | 7752 | 1615 | | 1039 | 148 | |  | |
| Deaths | | 478 | 151 | | 73 | 8 | |  | |
| Person-years | | 117,188 | 24,116 | | 14,360 | 2,272 | |  | |
| Mortality rate 10^-3^ | | 4.08 | 6.26 | | 5.08 | 3.52 | |  | |
| Sex-age-adjusted HR (95% CI) | | 1.00 (Ref) | 1.11 (0.92 - 1.34)^a^ | | 0.90 (0.71 - 1.15) | 0.49 (0.27 - 0.91)^b^ | | ^a^p = 0.27 ^b^p = 0.10 | |
| MV-adjusted HR (95% CI) | | 1.00 (Ref) | 1.11 (0.91 - 1.35)^a^ | | 0.94 (0.71 - 1.26) | 0.54 (0.28 - 1.04)^b^ | | ^a^p = 0.29 ^b^p = 0.07 | |
| *MV-adjusted HR (95% CI) for wine vs. no wine*  *only among good MedDiet compliers:* | | | | | **1 (ref.)** | **0.48 (0.22 - 1.05)** | |  | |
|  | | **Cumulative total ethanol intake (g/d)** | | | | | |  | |
|  | **Non drinkers**  **0** | | | **Light**  **(>0 & <5)** | **Moderate**  **(5-25 women, 10-50 men)** | | **Heavy**  **(>25 women, >50 men)** | |  |
| Deaths | 127 | | | 326 | 234 | | 23 | |  |
| Person-years | 27,486 | | | 77,002 | 50,067 | | 3,382 | |  |
| Mortality rate 10^-3^ | 3.62 | | | 4.23 | 4.67 | | 6.80 | |  |
| Sex-age-adjusted HR (95% CI) | 1.23 (0.99 - 1.53) | | | 1.00 (Ref) | 1.29 (1.09 - 1.53) | | 2.24 (1.46 - 3.43) | |  |
| MV-adjusted HR (95% CI) | 1.18 (0.94 - 1.49) | | | 1.00 (Ref) | 1.27 (1.07 - 1.52) | | 1.85 (1.18 - 2.90) | |  |
| **CARDIOVASCULAR DISEASE (up to 22 years follow-up)** | | | | | | | | | |
|  | | **Cumulative average of MEDAS with or without wine** | | | | | |  | |
| **Cumulative average of MEDAS (without wine)** | | **Low MedDiet compliance**  **(0 to 8)** | | | **High MedDiet compliance**  **(9 to 13)** | | | **p value**  **(wine vs. no wine)** | |
| **Wine point at baseline** | | **No wine** | **Adding wine** | | **No wine** | **Adding wine** | |  | |
| **n** | | 7566 | 1548 | | 1000 | 138 | |  | |
| Incident cases of CVD | | 215 | 73 | | 28 | 8 | |  | |
| Person-years | | 114,685 | 23,255 | | 13,901 | 2,110 | |  | |
| Incidence rate 10^-3^ | | 1.87 | 3.14 | | 2.01 | 3.79 | |  | |
| Sex-age-adjusted HR (95% CI) | | 1.00 (Ref) | 1.18 (0.90 - 1.53)^a^ | | 0.88 (0.60 - 1.30) | 1.24 (0.61 - 2.52)^b^ | | ^a^p = 0.23 ^b^p = 0.31 | |
| MV-adjusted HR (95% CI) | | 1.00 (Ref) | 1.07 (0.81 - 1.42)^a^ | | 0.77 (0.49 - 1.22) | 1.45 (0.74 - 2.83)^b^ | | ^a^p = 0.64 ^b^p = 0.10 | |
| *MV-adjusted HR (95% CI) for wine vs. no wine*  *only among good MedDiet compliers:* | | | | | **1 (ref.)** | **2.56 (0.84 - 7.84)** | |  | |
|  | | **Cumulative total ethanol intake (g/d)** | | | | | |  | |
|  | **Non drinkers**  **0** | | | **Light**  **(>0 & <5)** | **Moderate**  **(5-25 women, 10-50 men)** | | **Heavy**  **(>25 women, >50 men)** | |  |
| Cases | 41 | | | 167 | 110 | | 6 | |  |
| Person-years | 26,809 | | | 75,280 | 48,571 | | 3,291 | |  |
| Incidence rate 10^-3^ | 1.53 | | | 2.22 | 2.26 | | 1.82 | |  |
| Sex-age-adjusted HR (95% CI) | 0.93 (0.65 - 1.33) | | | 1.00 (Ref) | 1.27 (1.00 - 1.61) | | 1.38 (0.59 - 3.20) | |  |
| MV-adjusted HR (95% CI) | 0.90 (0.62 - 1.30) | | | 1.00 (Ref) | 1.12 (0.87 - 1.45) | | 0.93 (0.37 - 2.34) | |  |

MEDAS: Mediterranean Diet Adherence Screener. MedDet: Mediterranean diet. HR: Hazard Ratio. MV: multivariable model with robust estimators of variance, adjusted for sex, age (underlying time variable, and strata for decades), body mass index (adding a quadratic term), physical activity, years of university studies, smoking status, smoking pack-years, marital status, prevalence of depression, diabetes, hypertension and cancer (also for prevalence of cardiovascular disease in models for total death), consumption of other alcoholic beverages (excluding wine). Stratified by sex, year of entry to the cohort and quartiles of total energy intake.
