# Supplementary material for: Wine consumption, Mediterranean diet, and cardiovascular risk in two Spanish cohorts
Source: Eur Heart J. 2026 Feb 11;47(27):3591–606. doi: 10.1093/eurheartj/ehaf1081 (PMC13364079; doi:10.1093/eurheartj/ehaf1081)
Supplement: ehaf1081_Supplementary_Data [file ehaf1081_supplementary_data.zip › Supplementary Table 5.docx]

**Supplementary Table 5**. Hazard ratios (HRs) and linear trend tests for cardiovascular disease and long-term all-cause mortality in the PREDIMED trial, according to joint categories of the Mediterranean Diet Adherence Screener (MEDAS) score and attainment of the wine point, using repeated measurements of both exposures and applying alternative options for the selection of confounders.

**CARDIOVASCULAR DISEASE during the PREDIMED trial (4.8 y follow-up)**

|  | **MEDAS** | | | |  |
| --- | --- | --- | --- | --- | --- |
|  | **Low MedDiet compliance (<=9)** | | **High MedDiet compliance**  **(>9)** | |  |
| **Wine point** | **No wine** | **Adding wine** | **No wine** | **Adding wine** |  |
| Number of cardiovascular events | 113 | 59 | 80 | 36 | **p (linear trend test)** |
| Crude HR | 1 (ref.) | 1.11 (0.81 - 1.52) | 0.75 (0.56 - 1.01) | 0.68 (0.47 - 0.99) | 0.011 |
| Age-, sex-adjusted HR | 1 (ref.) | 0.83 (0.60 - 1.16) | 0.78 (0.59 - 1.04) | 0.51 (0.35 - 0.76) | 0.001 |
| Minimally MV-adjusted HR (95% CI)* | 1 (ref.) | 0.85 (0.61 - 1.19) | 0.74 (0.55 - 0.99) | 0.49 (0.33 - 0.73) | <0.001 |
| Moderately MV-adjusted HR (95% CI)** | 1 (ref.) | 0.82 (0.58 - 1.16) | 0.73 (0.54 - 0.98) | 0.47 (0.32 - 0.70) | <0.001 |
| Maximally MV-adjusted HR (95% CI)*** | 1 (ref.) | 0.85 (0.60 - 1.21) | 0.84 (0.61 - 1.15) | 0.55 (0.36 - 0.83) | 0.007 |

**ALL-CAUSE MORTALITY during expanded follow-up of PREDIMED trial (17 y follow-up)**

|  | **MEDAS** | | | |  |
| --- | --- | --- | --- | --- | --- |
|  | **Low MedDiet compliance** | | **High MedDiet compliance** | |  |
| **Wine point** | **No wine** | **Adding wine** | **No wine** | **Adding wine** |  |
| Number of deaths | 680 | 303 | 646 | 301 | **p (linear trend test)** |
| Crude HR | 1 (ref.) | 1.13 (0.98 - 1.29) | 0.74 (0.67 - 0.83) | 0.78 (0.68 - 0.89) | <0.001 |
| Age-, sex-adjusted HR | 1 (ref.) | 0.98 (0.84 - 1.13) | 0.81 (0.73 - 0.91) | 0.70 (0.61 - 0.80) | <0.001 |
| Minimal MV-adjusted HR (95% CI)* | 1 (ref.) | 0.97 (0.84 - 1.12) | 0.80 (0.71 - 0.89) | 0.71 (0.61 - 0.82) | <0.001 |
| Moderate MV-adjusted HR (95% CI)** | 1 (ref.) | 0.98 (0.84 - 1.13) | 0.81 (0.72 - 0.90) | 0.71 (0.61 - 0.82) | <0.001 |
| Maximum MV-adjusted HR (95% CI)*** | 1 (ref.) | 0.95 (0.82 - 1.10) | 0.77 (0.68 - 0.87) | 0.67 (0.57 - 0.78) | <0.001 |

* Multivariable model, with robust estimators of variance adjusted for age, sex and major cardiovascular risk factors (smoking, diabetes, hypertension, dyslipidemia) with stratification by site.

** Multivariable model, with robust estimators of variance adjusted for age, sex, smoking, diabetes, hypertension, dyslipidemia, total energy intake, body mass index and educational level (5 categories), with stratification by site.

*** Multivariable model, with robust estimators of variance adjusted for age, smoking, diabetes, hypertension, dyslipidemia, physical activity, waist-to-height ratio, body mass index (including a quadratic term), total energy intake, fruit consumption, vegetable consumption, and dietary fiber intake. Stratified according to site, sex, educational level (five categories) and randomized arm of the trial.

Linear trend tests were always applied to a 4-category classification combining compliance with the 13-item MEDAS and the wine point (poor MedDiet complier and no wine point, code=0; poor MedDiet complier and attainment of the wine point, code=1; good MedDiet complier  and no wine point, code=2; good MedDiet complier and attainment of the wine point, code=3). Linear trend tests across these 4 categories were conducted using these 4 values as a continuous variable.
