# Supplementary material for: Wine consumption, Mediterranean diet, and cardiovascular risk in two Spanish cohorts
Source: Eur Heart J. 2026 Feb 11;47(27):3591–606. doi: 10.1093/eurheartj/ehaf1081 (PMC13364079; doi:10.1093/eurheartj/ehaf1081)
Supplement: ehaf1081_Supplementary_Data [file ehaf1081_supplementary_data.zip › Supplementary Table 6.docx]

**Supplementary Table 6**. Multivariable adjusted hazard ratios (HRs) with 95% confidence intervals (CI) for all-cause mortality in the long-term follow-up of PREDIMED trial according to joint categories of three variables (as graphically shown in Figure 3): random allocation (Mediterranean diet or control), cumulative average adherence to the Mediterranean Diet (according to the 13.item MEDAS score: <=9 vs. >9) and attainment of the wine point, using repeated measurements of these last two exposures (cumulative averages).

**ALL-CAUSE MORTALITY (up to 17 y follow-up)**

|  | **MEDAS** | | | |
| --- | --- | --- | --- | --- |
|  | **Low MedDiet adherence**  **(<=9)** | | **High MedDiet adherence**  **(>9)** | |
| **Wine point** | **No wine** | **Adding wine** | **No wine** | **Adding wine** |
| **RANDOMIZED TO ANY OF THE TWO MEDITERRANEAN DIET GROUPS** | | | | |
| Number of deaths | 327 | 165 | 514 | 250 |
| Person-years | 15932 | 7396 | 29792 | 14401 |
| MV-adjusted HR (95% CI) | 1 (ref.) | 0.92 (0.75 - 1.14) | 0.72 (0.62 - 0.83) | 0.61 (0.51 - 0.73) |
| **RANDOMIZED TO CONTROL (LOW-FAT) GROUP** | | | | |
| Number of deaths | 353 | 138 | 132 | 51 |
| Person-years | 16428 | 6158 | 7422 | 2560 |
| MV-adjusted HR (95% CI) | 1.14 (0.92 – 1.42) | 1.11 (0.86 - 1.44) | 1.02 (0.83 - 1.26) | 1.00 (0.73 - 1.38) |

MV: multivariable model, with robust estimators of variance adjusted for age, smoking, diabetes, hypertension, dyslipidemia, physical activity, waist-to-height ratio, body mass index (including a quadratic term), total energy intake, fruit consumption, vegetable consumption, and dietary fiber intake. All the models were stratified according to site, sex, educational level (five categories) and randomized arm of the trial.
