# Supplementary material for: Wine consumption, Mediterranean diet, and cardiovascular risk in two Spanish cohorts
Source: Eur Heart J. 2026 Feb 11;47(27):3591–606. doi: 10.1093/eurheartj/ehaf1081 (PMC13364079; doi:10.1093/eurheartj/ehaf1081)
Supplement: ehaf1081_Supplementary_Data [file ehaf1081_supplementary_data.zip › Supplementary table 7.docx]

**Supplementary Table 7**. Multivariable-adjusted hazard ratios (HRs) for all-cause mortality during the extended follow-up of the PREDIMED trial (excluding former drinkers and including only participants aged >=60 years) and in the SUN cohort (including only participants aged >40 years), according to cumulative exposures to total alcohol intake using the same classification criteria as Ortolá et al^33^. Mortality outcomes were assessed over up to 17 years of follow-up in PREDIMED and up to 22 years in SUN.

**LONG-TERM MORTALITY IN THE PREDIMED TRIAL**

**(in participants>=60 years, excluding former drinkers)**

|  | **Cumulative average of total ethanol intake (g/d) during each year of the trial** | | | | |
| --- | --- | --- | --- | --- | --- |
|  | **No** | **Occasional** | **Light** | **Moderate** | **Heavy** |
|  | **Abstention (0)** | **>0 to <=2.86 g/d** | **>2.86 to 20 g/d (men)**  **>2.86 to 10 g/d (women)** | **>20 to 40 g/d (men)**  **>10 to 20 g/d (women)** | **> 40 g/d (men)**  **> 20 g/d (women)** |
| Person-years | 21,609 | 23,015 | 24,728 | 11,753 | 3,528 |
| Deaths | 471 | 434 | 522 | 244 | 70 |
| Age-, sex-adjusted HR (95% CI) | – | 1 (ref.) | 0.95 (0.82 – 1.09) | 0.99 (0.84 -1.17) | 1.09 (0.84 -1.42) |
| MV-adjusted HR (95% CI)^1^ | – | 1 (ref.) | 1.00 (0.87 – 1.16) | 1.02 (0.85 -1.22) | 1.06 (0.81 -1.40) |

| **LONG-TERM MORTALITY IN THE SUN COHORT** | | | | | |
| --- | --- | --- | --- | --- | --- |
|  | **Cumulative average of total ethanol intake (g/d)**  **using repeated measurements (at baseline and at 10-y follow-up)** | | | | |
|  | **No** | **Occasional** | **Light** | **Moderate** | **Heavy** |
|  | **Abstention (0)** | **>0 to <=2.86 g/d** | **>2.86 to 20 g/d (men)**  **>2.86 to 10 g/d (women)** | **>20 to 40 g/d (men)**  **>10 to 20 g/d (women)** | **> 40 g/d (men)**  **> 20 g/d (women)** |
| Person-years | 27,486 | 42,745 | 63,461 | 17,681 | 6,564 |
| Deaths | 127 | 159 | 285 | 97 | 42 |
| Age-, sex-adjusted HR (95% CI) | 1.26 (0.99 – 1.60) | 1 (ref.) | 1.08 (0.89 – 1.32) | 1.61 (1.25 – 2.06) | 1.94 (1.37 –2.75) |
| MV2-adjusted HR (95% CI)^2^ | 1.20 (0.93 – 1.55) | 1 (ref.) | 1.07 (0.87 – 1.32) | 1.50 (1.15 – 1.96) | 1.67 (1.14 –2.44) |

^1^ MV: multivariable, adjusted for age, smoking, diabetes, hypertension, dyslipidemia, physical activity, waist-to-height ratio, body mass index (including a quadratic term), total energy intake, fruit consumption, vegetable consumption, and dietary fiber intake, a robust variance estimator was used and the models were stratified according to site, sex, educational level (five categories) and randomized arm of the trial.

^2^ MV: multivariable model with robust estimators of variance, adjusted for sex, age (underlying time variable, and strata for decades), body mass index (adding a quadratic term), physical activity, years of university studies, smoking status, smoking pack-years, marital status, prevalence of depression, diabetes, hypertension and cancer (also for prevalence of cardiovascular disease in models for total death), consumption of other alcoholic beverages (excluding wine). Stratified by sex, year of entry to the cohort and quartiles of total energy intake.
