# Supplementary material for: Wine consumption, Mediterranean diet, and cardiovascular risk in two Spanish cohorts
Source: Eur Heart J. 2026 Feb 11;47(27):3591–606. doi: 10.1093/eurheartj/ehaf1081 (PMC13364079; doi:10.1093/eurheartj/ehaf1081)
Supplement: ehaf1081_Supplementary_Data [file ehaf1081_supplementary_data.zip › Supplementary Table 8.docx]

**Supplementary Table 8**. Multivariable-adjusted hazard ratios (HRs) for all-cause mortality during the extended follow-up of the PREDIMED trial (2003-2020), according to cumulative exposures to total alcohol intake merging former drinkers, moderate drinkers and heavy drinkers (all together as the reference category) to assess the risk for the merged categories of occasional and light drinkers. Mortality outcomes were assessed over up to 17 years of follow-up.

|  | **Former drinker** | **Moderate drinker >20 to 40 g/d (men)**  **>10 to 20 g/d (women)** | **Heavy drinker**  **> 40 g/d (men)**  **> 20 g/d (women)** | **Occasional**  **drinker**  **(>0 to <=2.86 g/d)** | **Light drinker**  **>2.86 to 20 g/d (men)**  **2.86 to 10 g/d (women)** |
| --- | --- | --- | --- | --- | --- |
|  | **Exposure group** | | | **Reference group** | |
| Person-years | 22,640 | | | 54,209 | |
| Deaths | 433 | | | 1009 | |
| Age-, sex-adjusted HR (95% CI) | 1.09 (0.97 – 1.22) | | | 1 (ref.) | |
| MV-adjusted HR (95% CI) | 1.08 (0.96 – 1.22) | | | 1 (ref.) | |
| MV-adjusted HR (95% CI) excluding former drinkers^1^ (n=325) | 1.02 (0.89 – 1.17) | | | 1 (ref.) | |
| MV-adjusted HR (95% CI) excluding former drinkers^2^ (n=1389) | 1.01 (0.88 – 1.15) | | | 1 (ref.) | |
| MV-adjusted HR (95% CI) in participants >=60 years and excluding former drinkers^2^ (n=1310) | 1.01 (0.88 – 1.16) | | | 1 (ref.) | |

*^1^ Former drinkers were defined as participants who reported to have had their last consumption of alcoholic beverages during the year earlier or even earlier before entering the PREDIMED trial (n=325).*

*^2^ Former drinkers were defined as participants who acknowledged any previous consumption of alcoholic beverages in any time point before entering the trial but reported no alcohol consumption at all when they filled in the FFQ (n=1389).*
