# Supplementary material for: Wine consumption, Mediterranean diet, and cardiovascular risk in two Spanish cohorts
Source: Eur Heart J. 2026 Feb 11;47(27):3591–606. doi: 10.1093/eurheartj/ehaf1081 (PMC13364079; doi:10.1093/eurheartj/ehaf1081)
Supplement: ehaf1081_Supplementary_Data [file ehaf1081_supplementary_data.zip › Supplementary Table 9.docx]

**Supplementary Table 9**. Burden of previous diseases (% at baseline) according to alcohol drinking categories in the PREDIMED trial.

| **% with previous disease at baseline^1^** | **Never drinker** | **Former drinker^2^** | **Occasional drinker**  **>0 to <=2.86 g/d** | **Light drinker**  **>2.86 to 20 g/d (men)**  **2.86 to 10 g/d (women)** | **Moderate drinker**  **>20 to 40 g/d (men)**  **>10 to 20 g/d (women)** | **Heavy drinker**  **> 40 g/d (men)**  **> 20 g/d (women)** | **Crude**  **p value** | **Age- and sex-adjusted**  **p value** |
| --- | --- | --- | --- | --- | --- | --- | --- | --- |
| Depression | **24.2*** (22.6) | 17.2 (**25.0***) | 19.0 | 12.4 | 13.1 | 12.1 | <0.001 | <0.001 |
| Pulmonary embolism | 0.6 (0.8) | 0.3 (0.4) | 0.5 | 0.3 | 0.6 | 0.0 | 0.41 | 0.83 |
| Aortic aneurism | 0.1 (0.1) | 0.0 (0.1) | 0.2 | 0.4 | 0.1 | 0.2 | 0.48 | 0.79 |
| Heart failure | 0.8 (1.0) | 0.0 (0.4) | 0.6 | 0.5 | 0.5 | 0.7 | 0.61 | 0.88 |
| Deep venous thrombosis | 1.4 (1.6) | 0.9 (1.2) | 1.5 | 0.8 | 0.6 | 0.4 | 0.08 | 0.26 |
| Bone fracture | 17.0 (15.5) | 13.9 (18.0) | 17.9 | 19.6 | 20.4 | **20.9*** | 0.013 | 0.04 |
| Retinopathy | 3.2 (2.8) | 1.5 (3.5) | 2.1 | 2.5 | 2.1 | 1.5 | 0.07 | 0.10 |
| CVD different from CHD or stroke | 3.1 (2.2) | 2.2 (3.8) | 2.3 | 2.4 | 2.1 | 2.2 | 0.39 | 0.50 |
| Kidney disease | 2.9 (2.2) | 2.5 (3.5) | 2.8 | 2.8 | 1.9 | 2.4 | 0.60 | 0.31 |
| Chronic lung disease | 3.9 (**5.4***) | 4.6 (2.7) | 4.4 | 5.4 | 5.4 | 6.4 | 0.09 | 0.86 |
| Sleep apnea | 1.2 (1.2) | 0.3 (1.2) | 2.3 | 2.37 | 1.8 | **2.42*** | 0.012 | 0.10 |
| Parkinson disease | 0.3 (0.3) | 0.3 (0.4) | 0.2 | 0.1 | 0.4 | 0.0 | 0.55 | 0.48 |
| Cancer | 3.1 (2.2) | 2.8 (3.9) | 3.0 | 2.6 | 3.1 | 2.4 | 0.92 | 0.91 |
| ***>1 disease*** | **13.5*** (12.3) | 7.7 (**13.8***) | 10.4 | 10.0 | 10.0 | 9.7 | <0.001 | 0.02 |
| ***>1 disease (excluding depression)*** | 5.9 (4.6) | 2.8 (6.7) | 5.0 | 6.3 | 6.2 | 5.9 | 0.16 | 0.16 |
| ***>2 diseases*** | **2.7*** (1.7) | 1.2 (**3.5***) | 2.3 | 2.4 | 1.9 | 2.6 | 0.51 | 0.47 |
| ***>2 diseases (excluding depression)*** | 0.87 (0.60) | 0.00 (1.01) | 0.78 | 0.87 | 0.90 | 1.54 | 0.37 | 0.81 |

CVD: Cardiovascular disease; CHD: Coronary Heart Disease.

^1^ Percentages shown between brackets in the first two columns correspond to using a wide definition of former drinkers meaning participants who acknowledged any previous consumption of alcoholic beverages at any time point before entering the trial and reported no alcohol consumption at all when they filled in the FFQ (n=1389).

^2^ The first percentages shown in this column correspond to former drinkers defined as participants who reported to have had their last consumption of alcoholic beverages during the year earlier or even earlier before entering the PREDIMED trial (n=325).
